# Group IIA secreted phospholipase A_2_ controls skin carcinogenesis and psoriasis by shaping the gut microbiota

**DOI:** 10.1172/jci.insight.152611

**Published:** 2022-01-25

**Authors:** Yoshimi Miki, Yoshitaka Taketomi, Yuh Kidoguchi, Kei Yamamoto, Kazuaki Muramatsu, Yasumasa Nishito, Jonguk Park, Koji Hosomi, Kenji Mizuguchi, Jun Kunisawa, Tomoyoshi Soga, Eric Boilard, Siddabasave Gowda B. Gowda, Kazutaka Ikeda, Makoto Arita, Makoto Murakami

**Affiliations:** 1 Laboratory of Microenvironmental and Metabolic Health Science, Center for Disease Biology and Integrative Medicine, Graduate School of Medicine, The University of Tokyo (UTokyo), Tokyo, Japan.; 2 Lipid Metabolism Project, Tokyo Metropolitan Institute of Medical Science (TMIMS), Tokyo, Japan.; 3 School of Science and Engineering, Tokyo Denki University, Saitama, Japan.; 4 Division of Bioscience and Bioindustry, Tokushima University, Tokushima, Japan.; 5 Center for Basic Technology Research, TMIMS, Tokyo, Japan.; 6 Artificial Intelligence Center for Health and Biomedical Research and; 7 Laboratory of Vaccine Materials, Center for Vaccine and Adjuvant Research and Laboratory of Gut Environmental System, National Institutes of Biomedical Innovation, Health and Nutrition (NIBIOHN), Ibaraki, Osaka, Japan.; 8 Institute for Protein Research, Osaka University, Suita, Osaka, Japan.; 9 Institute for Advanced Biosciences, Keio University, Tsuruoka, Yamagata, Japan.; 10 Centre de Recherche du CHU de Québec, Centre de Recherche Arthrite de l’Université Laval, Department of Microbiology and Immunology, Québec, Canada.; 11 Laboratory for Metabolomics, RIKEN Center for Integrative Medical Sciences, Yokohama, Kanagawa, Japan.; 12 Division of Physiological Chemistry and Metabolism, Graduate School of Pharmaceutical Sciences, Keio University, Tokyo, Japan.

**Keywords:** Inflammation, Microbiology, Molecular pathology, Mouse models, Skin

## Abstract

Besides promoting inflammation by mobilizing lipid mediators, group IIA secreted phospholipase A_2_ (sPLA_2_-IIA) prevents bacterial infection by degrading bacterial membranes. Here, we show that, despite the restricted intestinal expression of sPLA_2_-IIA in BALB/c mice, its genetic deletion leads to amelioration of cancer and exacerbation of psoriasis in distal skin. Intestinal expression of sPLA_2_-IIA is reduced after treatment with antibiotics or under germ-free conditions, suggesting its upregulation by gut microbiota. Metagenome, transcriptome, and metabolome analyses have revealed that sPLA_2_-IIA deficiency alters the gut microbiota, accompanied by notable changes in the intestinal expression of genes related to immunity and metabolism, as well as in the levels of various blood metabolites and fecal bacterial lipids, suggesting that sPLA_2_-IIA contributes to shaping of the gut microbiota. The skin phenotypes in *Pla2g2a*^–/–^ mice are lost (a) when they are cohoused with littermate WT mice, resulting in the mixing of the microbiota between the genotypes, or (b) when they are housed in a more stringent pathogen-free facility, where *Pla2g2a* expression in WT mice is low and the gut microbial compositions in both genotypes are nearly identical. Thus, our results highlight a potentially new aspect of sPLA_2_-IIA as a modulator of gut microbiota, perturbation of which affects distal skin responses.

## Graphical abstract

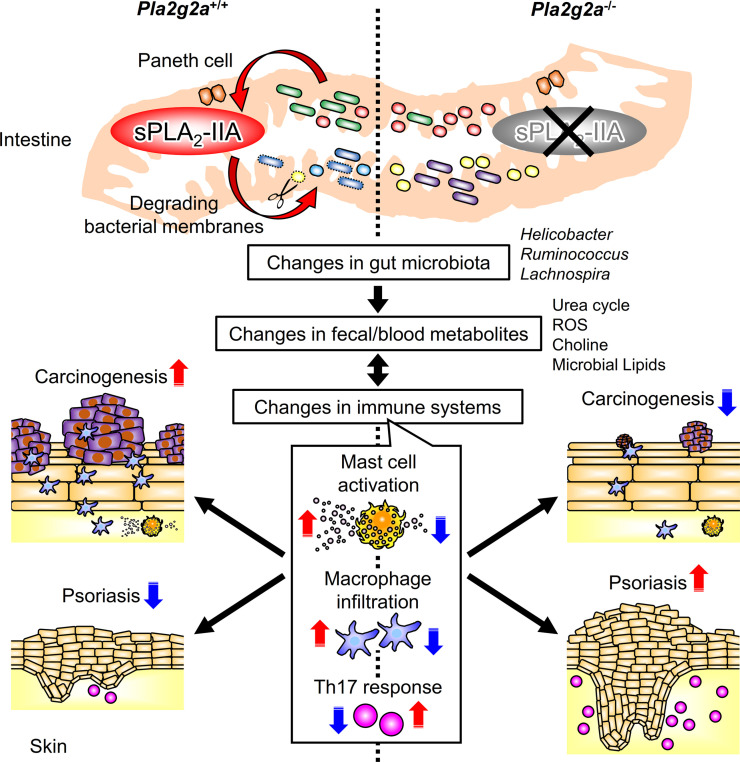

## Introduction

The gut microbiota present on the epithelial barriers comprises approximately 3 × 10^13^ microbial cells, most of which exhibit commensalism with the host. Imbalance of gut microbial composition, known as dysbiosis, is caused by many factors, including host genetics and lifestyle (1). The microbiota influences the physiological functions of the host, ranging from the maintenance of local gut homeostasis to the systemic regulation of metabolism, immunity, hematopoiesis, and neurological functions (2, 3). Over the last few decades, there has been a marked increase in the prevalence of colitis, allergy, and metabolic disease in developed countries in response to dysbiosis caused by low consumption of dietary fiber and/or high consumption of sugar, fat, or salt (4, 5). The microbiota is also involved in the initiation, progression, and dissemination of cancer, both at epithelial barriers and in sterile tissues (6, 7). Various microbial metabolites such as tryptophan derivatives, vitamins, secondary bile acids, and short-chain fatty acids have profound influences on host responses (8–10). Genetic deletion of several host factors involved in epithelial barrier or innate/adaptive immunity causes dysbiosis, resulting in altered susceptibility to various diseases, which can be transferred to WT mice by cohousing or fecal transfer (11, 12). Indeed, transfer of feces from healthy donors to individuals with metabolic syndrome has been shown to alter the gut microbiota of the recipients, leading to improved insulin sensitivity (13).

The secreted phospholipase A_2_ (sPLA_2_) family is a group of extracellular lipolytic enzymes that contains 11 isoforms in mammals (14). Recent studies using mice gene-manipulated for individual sPLA_2_s have revealed their diverse roles in various biological events by driving specific lipid pathways in given microenvironments (14, 15). sPLA_2_ group IIA (sPLA_2_-IIA), an inflammatory sPLA_2_ that is induced in various human tissues following inflammatory stimuli (15, 16), has been implicated in the exacerbation of sterile inflammation through production of lipid mediators by acting on phospholipids in extracellular vesicles (17, 18). In addition, sPLA_2_-IIA efficiently degrades bacterial membranes, thereby playing a protective role against bacterial infection as a bactericidal sPLA_2_ (19). Indeed, sPLA_2_-IIA kills Gram-positive bacteria (e.g., *Staphylococcus aureus* and *Bacillus anthracis*) at 1–100 ng/mL, and also Gram-negative bacteria (e.g., *E. coli*) at 100–500 ng/mL with the aid of cofactors such as bacterial permeability-increasing protein (BPI) (16, 20, 21). Through this bactericidal activity, Tg mice overexpressing human sPLA_2_-IIA (*PLA2G2A^TGN^*) on a C57BL/6 background show resistance to pneumonia and septic shock after infection with Gram-positive or -negative bacteria (21, 22). The highly cationic nature of sPLA_2_-IIA is critical for its penetration across the bacterial wall and, thereby, hydrolysis of bacterial membranes (23, 24).

Since sPLA_2_-IIA is abundantly expressed in the intestine — particularly in Paneth cells, which secrete a cocktail of antimicrobial peptides (25, 26) — it has been anticipated that the enzyme may have some impacts on commensal microbiota in the gut. Indeed, the concentration of sPLA_2_-IIA protein in the small intestine of BALB/c mice is estimated to be ~2000 ng/mg tissue (27), which appears to be a high enough concentration to hydrolyze phospholipids in Gram-positive bacteria and even Gram-negative bacteria in cooperation with other antimicrobial peptides. However, this hypothesis has not yet been firmly confirmed because of the lack of a proper sPLA_2_-IIA–deficient mouse model until recently. The *Pla2g2a* gene (encoding sPLA_2_-IIA) is naturally disrupted in C57BL/6 and 129/Sv strains due to a frameshift mutation (28), which made it difficult to assess the precise functions of endogenous sPLA_2_-IIA in vivo using a standard KO strategy. Other mouse strains such as BALB/c, C3H, and DBA/1 have an intact *Pla2g2a* gene (28), but unlike the situation in human and rat, where sPLA_2_-IIA is expressed or induced in many tissues, its expression in these mouse strains is highly restricted to the intestine (25, 29). Recently, a BALB/c strain harboring a mutated *Pla2g2a* allele (*Pla2g2a*^–/–^ BALB/c mice) was obtained by backcrossing the mutated *Pla2g2a* allele in 129/Sv mice (30) or C57BL/6 mice (this study) onto a BALB/c background, making it possible to address the function of endogenous sPLA_2_-IIA. This mouse model is best suited for analyzing the role of endogenous sPLA_2_-IIA in the intestine, since the influence of sPLA_2_-IIA expressed in other tissues is negligible. In the present study using *Pla2g2a*^–/–^ BALB/c mice, we provide evidence that sPLA_2_-IIA indeed contributes to shaping of the gut microbiota, thereby having secondary impacts on cancer and psoriasis in distal skin. Our study has established a potentially novel mode of sPLA_2_ action in biological responses and adds this extracellular lipolytic enzyme family to a growing list of host factors that control gut microbiota.

## Results

### Pla2g2a^–/–^ BALB/c mice are protected from skin cancer.

**Figure 1:**
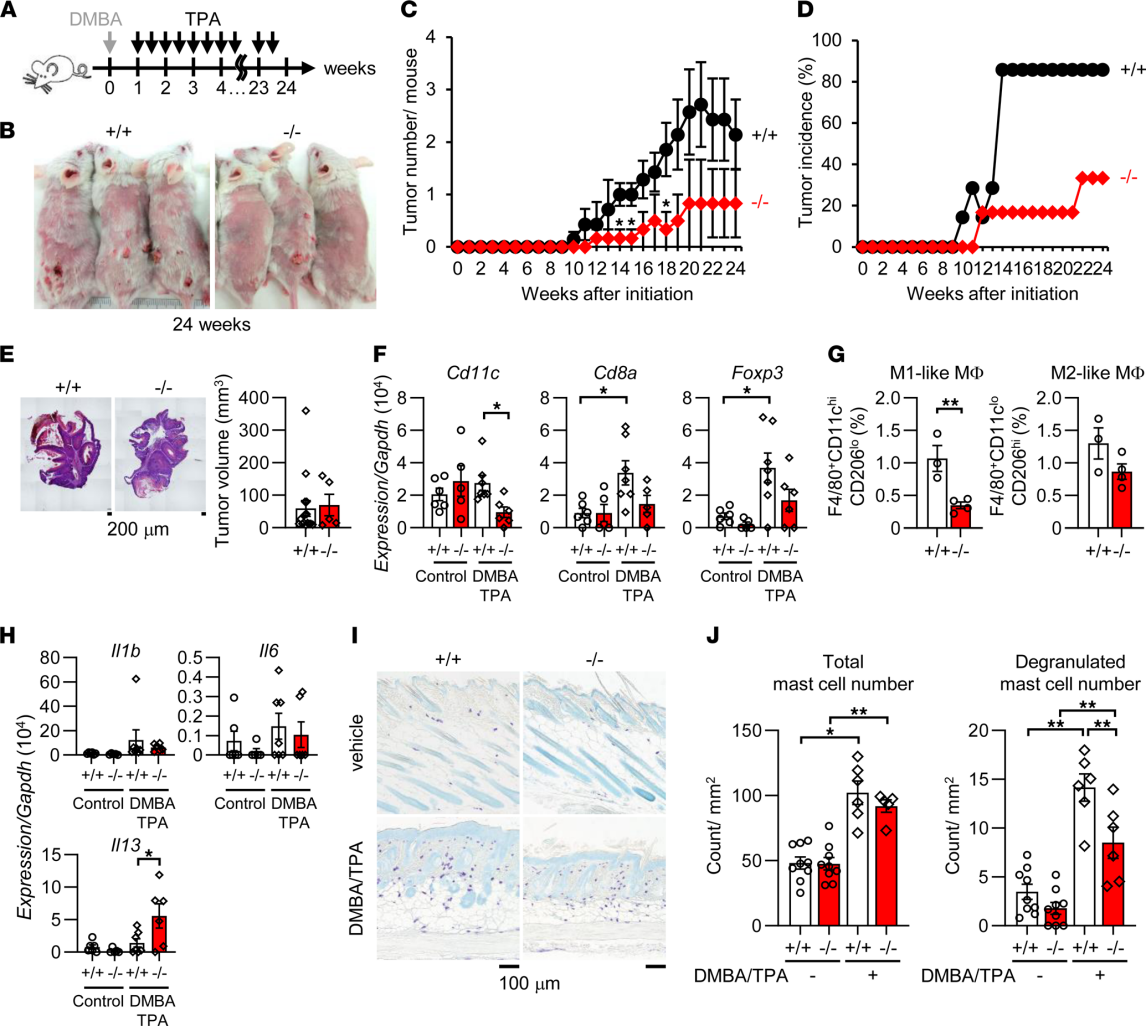
Altered skin carcinogenesis and PCA reaction in *Pla2g2a*^–/–^ mice. (**A**) The procedure for DMBA/TPA-induced skin carcinogenesis. (**B**) Representative photos at 24 weeks after DMBA/TPA treatment. (**C** and **D**) Time course of tumor number (**C**) and tumor incidence (**D**) in *Pla2g2a*^+/+^ and *Pla2g2a*^–/–^ mice after DMBA/TPA treatment (*n* = 6–7). (**E**) Representative photos of the tumor and tumor volume in *Pla2g2a*^+/+^ and *Pla2g2a*^–/–^ mice at 24 weeks (*n* = 5–15). (**F**) qPCR of several immune cell markers in the skin of *Pla2g2a*^+/+^ and *Pla2g2a*^–/–^ mice with or without DMBA/TPA treatment for 24 weeks (*n* = 5–7). (**G**) Flow cytometry of M1- and M2-like macrophages in the skin of *Pla2g2a*^+/+^ and *Pla2g2a*^–/–^ mice at 24 weeks (*n* = 3–4). (**H**) qPCR of cytokines in the skin of *Pla2g2a*^+/+^ and *Pla2g2a*^–/–^ mice with or without DMBA/TPA treatment at 24 weeks (*n* = 5–7). (**I**) Toluidine blue staining of skin sections from *Pla2g2a*^+/+^ and *Pla2g2a*^–/–^ mice with or without DMBA/TPA treatment at 24 weeks. Scale bars: 100 μm. (**J**) Counts of total and degranulated mast cells in **I** (*n* = 6–9). Data are shown as mean ± SEM; *P < 0.05, **P < 0.01. *P* values were calculated by Multiple *t* test using the Holm-Sidak method (**C**), Mann-Whitney *U* test (**E**), Student *t* test (**G**), and 1-way ANOVA (**F**, **H**, and **J**). Data are representative of 3 experiments (**B**–**E**) or from 1 experiment (**F**–**J**).

We previously showed that, when a model of skin carcinogenesis induced by 9,10-dimethylbenz(*a*)anthracene (DMBA) and 12-*O*-tetradecanoylphorbol-13-acetate (TPA) was applied to various sPLA_2_-deficient mice on a BALB/c background (a strain that is sensitive to this model) (Figure 1A), a lack of sPLA_2_-IID, which is expressed in lymphatic DCs and M2 macrophages, augments antitumor immunity through reduced mobilization of ω3 fatty acids (31), whereas a lack of sPLA_2_-IIF, which is expressed in epidermal keratinocytes, attenuates keratinocyte hyperproliferation through reduced mobilization of a specific lysophospholipid (32), thereby preventing skin carcinogenesis. Additionally, we found that the development of skin cancer was less marked in *Pla2g2a^–/–^* mice than in *Pla2g2a*^+/+^ mice (Figure 1B). Tumor multiplicity and incidence over time were delayed in *Pla2g2a*^–/–^ mice relative to *Pla2g2a*^+/+^ mice (Figure 1, C and D), although the volume of the tumors, once they had developed, was similar between the genotypes (Figure 1E). These results suggest that sPLA_2_-IIA deficiency prevents the initiation, rather than progression, of skin cancer.

Quantitative PCR (qPCR) of the skin revealed that the expression of *Cd11c* (a marker for M1 macrophages and DCs) was reduced in DMBA/TPA-treated *Pla2g2a*^–/–^ mice relative to *Pla2g2a*^+/+^ mice (Figure 1F). In agreement, flow cytometry demonstrated a significant reduction of M1-like macrophages (F4/80^+^CD11c^hi^CD206^lo^) and also a trend toward a reduction of M2-like macrophages (F4/80^+^CD11c^lo^CD206^hi^) in the skin of *Pla2g2a*^–/–^ mice relative to *Pla2g2a*^+/+^ mice (Figure 1G and Supplemental Figure 1; supplemental material available online with this article; https://doi.org/10.1172/jci.insight.152611DS1). The expression levels of *Cd8* (a cytotoxic T cell marker) and *Foxp3* (a Treg marker) were elevated in the DMBA/TPA-treated skin over control skin of *Pla2g2a*^+/+^ mice, whereas these responses were only modest in *Pla2g2a*^–/–^ mice (Figure 1F). Cutaneous expression of proinflammatory cytokines (*Il1b* and *Il6*) also showed a similar trend (Figure 1H). Expression of *Il13*, which is reportedly expressed in intraepidermal Vγ5Vδ1^+^ T cells and protective against skin carcinogenesis (33), was elevated in the skin of DMBA/TPA-treated *Pla2g2a*^–/–^ mice relative to that of *Pla2g2a*^+/+^ mice (Figure 1H), which might account, at least partly, for the amelioration of skin carcinogenesis by sPLA_2_-IIA deletion. Whereas DMBA/TPA treatment increased the number of dermal mast cells — which affect DMBA/TPA-induced skin carcinogenesis (34) — in both genotypes equally, degranulated mast cells were significantly fewer in *Pla2g2a*^–/–^ than in *Pla2g2a*^+/+^ mice (Figure 1, I and J). Thus, sPLA_2_-IIA deficiency in BALB/c mice attenuates skin carcinogenesis, with alterations in both inflammatory and regulatory arms of the immune response in DMBA/TPA-treated skin.

### Intestinal sPLA_2_-IIA expression is regulated by gut microbiota.

**Figure 2:**
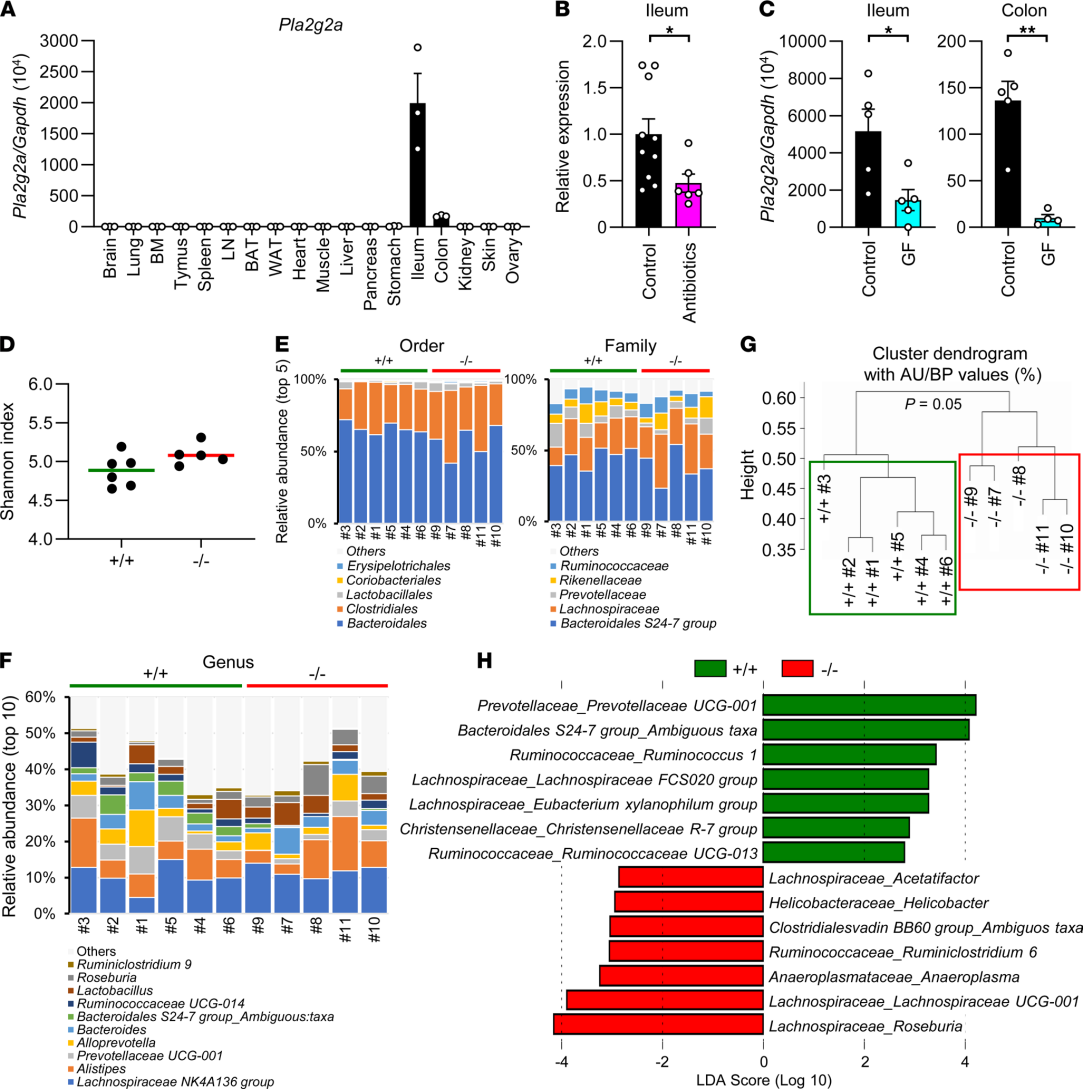
Genetic deletion of *Pla2g2a* alters gut microbiota. (**A**) qPCR of *Pla2g2a* in various tissues of BALB/c mice (*n* = 3). (**B** and **C**) Effects of antibiotic treatment (*n* = 6–10) (**B**) and germ-free (GF) conditions (*n* = 4–5) (**C**) on intestinal expression of *Pla2g2a*. Data are shown as mean ± SEM; *P < 0.05, **P < 0.01; Student *t* test. (**D**) Shannon bacterial diversity in the feces of *Pla2g2a*^+/+^ (*n* = 6) and *Pla2g2a*^–/–^ (*n* = 5) mice. Data are shown as medians. (**E** and **F**) Barcharts of bacterial compositions at the order and family (**E**), as well as genus (**F**) levels in the feces of *Pla2g2a*^+/+^ and *Pla2g2a*^–/–^ mice. (**G**) Hierarchical clustering with β-diversity of fecal microbiota at the genus level in *Pla2g2a*^+/+^ and *Pla2g2a*^–/–^ mice. (**H**) LEfSe analysis of fecal microbiota in *Pla2g2a*^+/+^ and *Pla2g2a*^–/–^ mice. Data are compiled from 1 (**A** and **C**) or 2 experiments (**B**) or representative of 2 experiments (**D**–**H**).

Reportedly, skin-specific mouse sPLA_2_-IIA Tg mice (*K14-Pla2g2a^TGN^*) showed increased skin carcinogenesis (35), and *PLA2G2A* knockdown in human skin squamous cell carcinoma reduced tumorigenesity (36). Beyond these skin-intrinsic actions of sPLA_2_-IIA in Tg mice and humans, however, the present finding that *Pla2g2a*^–/–^ mice displayed a skin phenotype was surprising, since it has been reported that sPLA_2_-IIA is expressed almost exclusively in Paneth cells in the small intestine of BALB/c mice (25, 26, 29). Indeed, we confirmed that *Pla2g2a* was abundantly expressed in the small intestine (jejunum = ileum > duodenum) and to a lesser extent in the large intestine (descending colon > ascending colon = cecum > rectum) of BALB/c mice (Figure 2A and Supplemental Figure 2, A and B). The expression levels of *Pla2g2a* in other tissues relative to the small and large intestines were nearly negligible (Figure 2A), with only trace expression being detected in several tissues (Supplemental Figure 2C). *Pla2g2a* expression was barely detectable in the skin (Supplemental Figure 2D) and was present at only a trace level in blood cells from *Pla2g2a*^+/+^, but not *Pla2g2a*^–/–^, mice regardless of DMBA/TPA treatment (Supplemental Figure 2E). Thus, we hypothesized that the skin phenotype observed in *Pla2g2a*^–/–^ mice might reflect a secondary effect resulting from some alterations in other tissues, possibly in the intestine where sPLA_2_-IIA is expressed abundantly.

Although expression of sPLA_2_-IIA is induced by proinflammatory stimuli such as LPS in various tissues of human, rat, and rabbit (37, 38), its expression in the intestine of BALB/c mice is not profoundly affected, even after LPS treatment (29). We thus speculated that the high steady-state expression of sPLA_2_-IIA in the intestine of BALB/c mice might be attributable to constitutive exposure to commensal microbiota in the lumen. In support of this hypothesis, *Pla2g2a* expression in the ileum and colon was reduced after treatment with antibiotics (Figure 2B) or under germ-free conditions (Figure 2C), suggesting that some microbial components derived from gut microbiota contribute to the high and constitutive expression of *Pla2g2a* in the intestine.

### sPLA_2_-IIA deficiency alters gut microbiota.

Considering the expression profile of sPLA_2_-IIA (Figure 2, A–C), we speculated that the lack of intestinal sPLA_2_-IIA would have some influence on gut microbiota, thereby secondarily affecting carcinogenesis in distal skin. To address this issue, we examined microbiota in the feces of *Pla2g2a*^+/+^ and *Pla2g2a*^–/–^ mice by high-throughput gene-sequencing analysis of bacterial 16S rRNA. To rule out the possibility of familial transmission that could affect the gut microbiota (39), we analyzed the feces from littermate *Pla2g2a*^+/+^ and *Pla2g2a*^–/–^ mice that had originated from the same *Pla2g2a*^+/−^ parents and had been housed separately by genotype after weaning. Taxonomically, bacteria are classified systematically in descending order from kingdom and then to phylum, class, order, family, genus, and species. Operational taxonomic unit (OTU) analysis showed that approximately 600 OTUs were present in both *Pla2g2a*^–/–^ and *Pla2g2a*^+/+^ mice, with no apparent difference in α-diversity determined by the Shannon index (Figure 2D). The absolute abundance (i.e., microbial load) of fecal microbiota and their relative abundance at the order and family levels did not differ significantly between the genotypes (Figure 2E). At the genus level, however, we observed notable differences in the relative abundance of several bacteria between *Pla2g2a*^+/+^ and *Pla2g2a*^–/–^ mice (Figure 2, F–H). Indeed, hierarchical clustering with β-diversity at the genus level resulted in separation of the microbiota into 2 groups — one consisting of the *Pla2g2a*^+/+^ cluster and the other consisting of the *Pla2g2a*^–/–^ cluster (Figure 2G) — suggesting that the composition of gut microbiota in *Pla2g2a*^–/–^ mice differs from that in *Pla2g2a*^+/+^ mice. Linear discriminant analysis effect size (LEfSe) analysis showed that several bacterial genera such as Gram-positive *Lachnospiraceae* and *Ruminococcaceae,* as well as Gram-negative *Prevotellaceae* and *Helicobacteraceae*, were differently distributed in *Pla2g2a*^–/–^ mice and *Pla2g2a*^+/+^ mice (Figure 2H). Evaluation of the gut microbiota by the random forest classification analysis also demonstrated that bacteria belonging to the *Ruminococcaceae*, *Lachnospiraceae*, and *Helicobacteraceae* families were reproducibly affected as notable variables in 2 independent sets of the experiment (Supplemental Figure 3). Reportedly, *Helicobacteraceae* exacerbates enteritis and gastric cancer (40, 41), while *Lachnospiraceae* and *Ruminococcaceae* have suppressive effects on colitis by producing butyrate (4, 42). These results suggest that sPLA_2_-IIA indeed contributes to shaping of the gut microbiota.

### Altered gut microbiota underlies the skin phenotypes in Pla2g2a^–/–^ mice.

**Figure 3:**
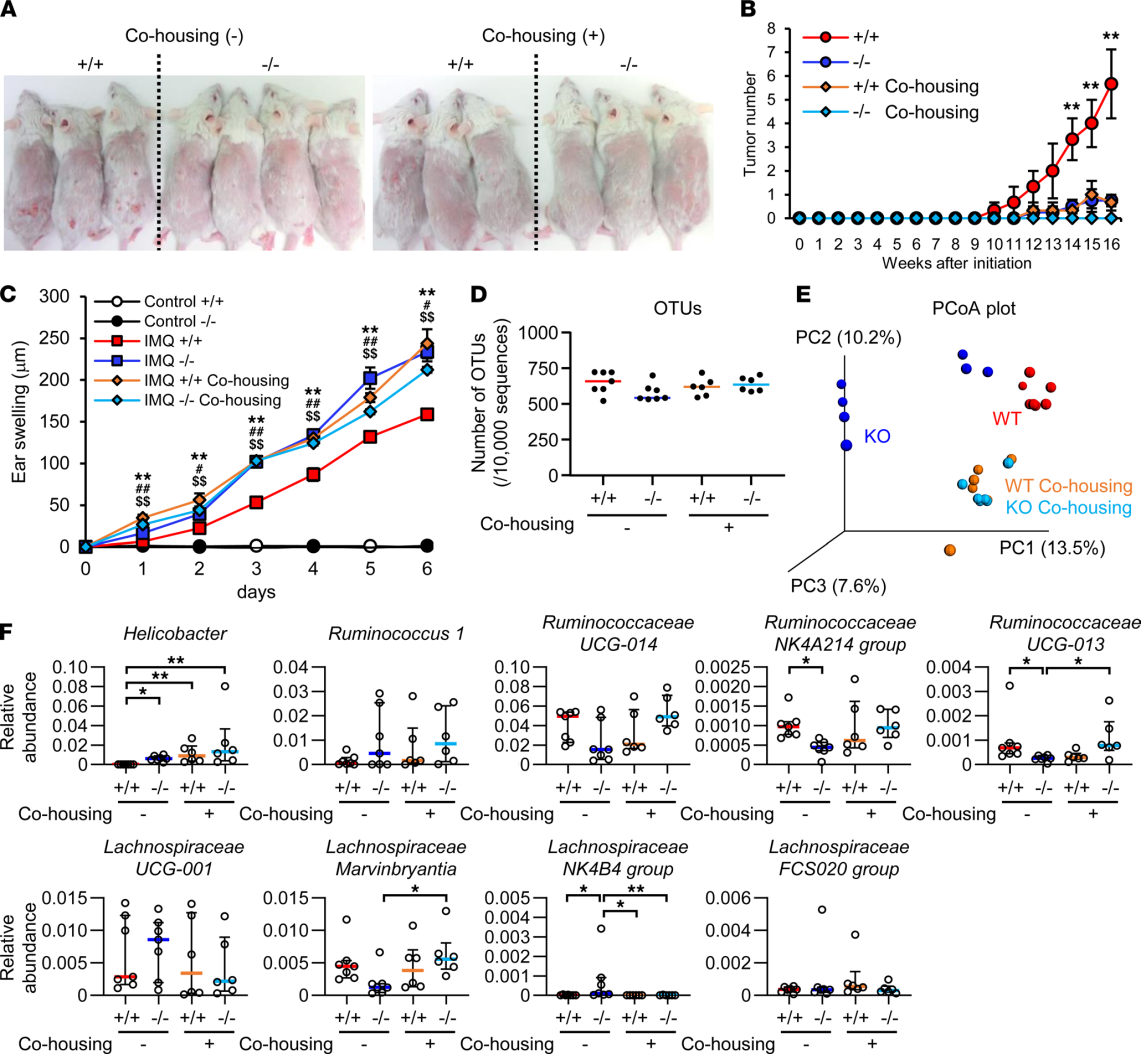
Altered skin phenotypes in *Pla2g2a*^–/–^ mice after cohousing with WT mice. (**A** and **B**) Representative photos at 16 weeks (**A**) and time course of skin tumor development (**B**) in DMBA/TPA-treated *Pla2g2a*^+/+^ and *Pla2g2a*^–/–^ mice with or without cohousing (*n* = 3–4). (**C**) IMQ-induced psoriasis in *Pla2g2a*^+/+^ and *Pla2g2a*^–/–^ mice with (*n* = 4–6) or without (*n* = 6–16) cohousing. In **B** and **C**, data are shown as mean ± SEM. **P < 0.01, cohousing (–) *Pla2g2a*^+/+^ versus cohousing (–) *Pla2g2a*^–/–^; ^#^*P* < 0.05, ^##^*P* < 0.01, cohousing (+) *Pla2g2a*^–/–^ versus cohousing (–) *Pla2g2a*^–/–^; and ^$$^*P* < 0.01, cohousing (+) *Pla2g2a*^+/+^ versus cohousing (–) *Pla2g2a*^–/–^ among IMQ-treated groups. (**D** and **E**) OTU analysis (**D**) and PCoA analysis based on UniFrac phylogenetic distances (**E**) of fecal microbiota in *Pla2g2a*^+/+^ and *Pla2g2a*^–/–^ mice with or without cohousing. (**F**) Relative abundance of specific bacteria in feces of *Pla2g2a*^+/+^ and *Pla2g2a*^–/–^ mice with or without cohousing. Values indicate median ± IQR. *P < 0.05, **P < 0.01 by Multiple *t* test using the Holm-Sidak method (**B** and **C**), Mann-Whitney *U* test (**D**), and Kruskal-Wallis test (**F**). Data are compiled from 2 experiments (**C**–**F**) or from 1 experiment (**A** and **B**).

To determine whether the altered microbiota observed in *Pla2g2a*^–/–^ mice would be responsible for the skin cancer phenotype (Figure 1), we conducted microbiota-transfer studies by cohousing both genotypes, leading to exchange of the microbiota through coprophagia (5, 11, 42). To this end, age- and sex-matched *Pla2g2a*^+/+^ and *Pla2g2a*^–/–^ mice were either housed in different cages after weaning (cohousing [–]) or housed in the same cages throughout life (cohousing [+]). Remarkably, after receiving DMBA/TPA, skin tumor development was markedly lower in *Pla2g2a*^−/−^ mice than in *Pla2g2a*^+/+^ mice in the cohousing (–) group (Figure 1, B and C), whereas cohoused *Pla2g2a*^+/+^ and *Pla2g2a*^−/−^ mice displayed low tumor susceptibility, similar to the response seen in single-housed *Pla2g2a*^−/−^ mice (Figure 3, A and B).

To further address whether cohousing would affect any other skin phenotype in *Pla2g2a*^−/−^ mice, we applied an imiquimod-induced (IMQ-induced) psoriasis model. Ectopic application of IMQ elicited more severe ear edema in *Pla2g2a*^−/−^ mice than in *Pla2g2a*^+/+^ mice in the cohousing (–) group, whereas cohoused *Pla2g2a*^+/+^ and *Pla2g2a*^−/−^ mice displayed a similar response (Figure 3C). Again, the psoriatic responses observed in cohoused *Pla2g2a*^+/+^ and *Pla2g2a*^–/–^ mice were similar to those observed in single-housed *Pla2g2a*^–/–^ mice. Thus, regardless of the amelioration or exacerbation of the disease models, cohousing allows the skin phenotypes in *Pla2g2a*^+/+^ mice to be similar to those seen in single-housed *Pla2g2a*^–/–^ mice.

Although the OTU analysis of fecal microbiota from these 4 groups (i.e., *Pla2g2a*^+/+^ and *Pla2g2a*^−/−^ mice with or without cohousing) revealed that the bacterial diversity was unaffected by genotypes and housing conditions (Figure 3D), principal coordinate analysis (PCoA) based on unique fraction metric (UniFrac) phylogenetic distances to probe the degree of similarity among the groups demonstrated differences in the clustering of single-housed *Pla2g2a*^+/+^ and *Pla2g2a*^–/–^ mice, whereas these 2 genotypes after cohousing displayed a distinct and common cluster (Figure 3E). Although fecal microbiota in single-housed *Pla2g2a*^–/–^ mice formed 2 clusters, likely due to a caging effect, they nonetheless showed a clear separation from those in single-housed *Pla2g2a*^+/+^ mice and also those in cohoused *Pla2g2a*^+/+^ and *Pla2g2a*^–/–^ mice. These results imply that the microbiota transferred from *Pla2g2a*^–/–^ mice affected disease susceptibility in *Pla2g2a*^+/+^ mice (and vice versa) under the cohousing (+) condition.

Among several hit bacteria (e.g., *Helicobacteraceae*, *Ruminococcaceae*, and *Lachnospiraceae*) identified by random forest analysis (Supplemental Figure 3), a particular bacterial species, *Helicobacter OTU 2564048*, tended to show a correlation with the skin phenotypes; its level was very low in single-housed *Pla2g2a*^+/+^ mice, but it was present more abundantly and equally in single-housed *Pla2g2a*^–/–^ mice and cohoused *Pla2g2a*^+/+^ and *Pla2g2a*^–/–^ mice (Figure 3F). *Ruminococcaceae* (*UCG-013* and *NK4A214 group*) and *Lachnospiraceae Marvinbryantia* displayed an opposite trend, although the correlation in the cohousing group was less obvious (Figure 3F). A BLAST search showed that *Helicobacter* *OTU 2564048* was highly related to *H*. *japonicus* strain *MIT 01-6451 NR_149210.1* (99%), *H*. *marmotae* strain *MIT 04-8589 GU9027.1* (98%), and *H*. *pylori* strain *MKF8 AP017358.1* (96%). The increased *Helicobacter* colonization in *Pla2g2a*^–/–^ mice appears to be compatible with a previous study demonstrating that *PLA2G2A^TGN^* mice were protected against *H*. *felis* infection (43). Although it is generally considered that *Helicobacter* is associated with gastrointestinal inflammation and cancer (40, 41), several lines of evidence suggest that *H*. *pylori*–positive individuals have lower risks of allergy than do *H*. *pylori*–negative individuals (44, 45). Although the causal relationship between the fecal abundance of a specific *Helicobacter* or other bacteria species and the skin phenotypes in *Pla2g2a*^–/–^ mice remains to be elucidated, our results nonetheless provide strong support for the notion that regulation of gut microbiota by sPLA_2_-IIA, a Paneth cell–derived antibacterial protein, is linked to altered susceptibility to skin carcinogenesis and psoriasis.

### Altered intestinal gene expression in Pla2g2a^–/–^ mice.

**Figure 4:**
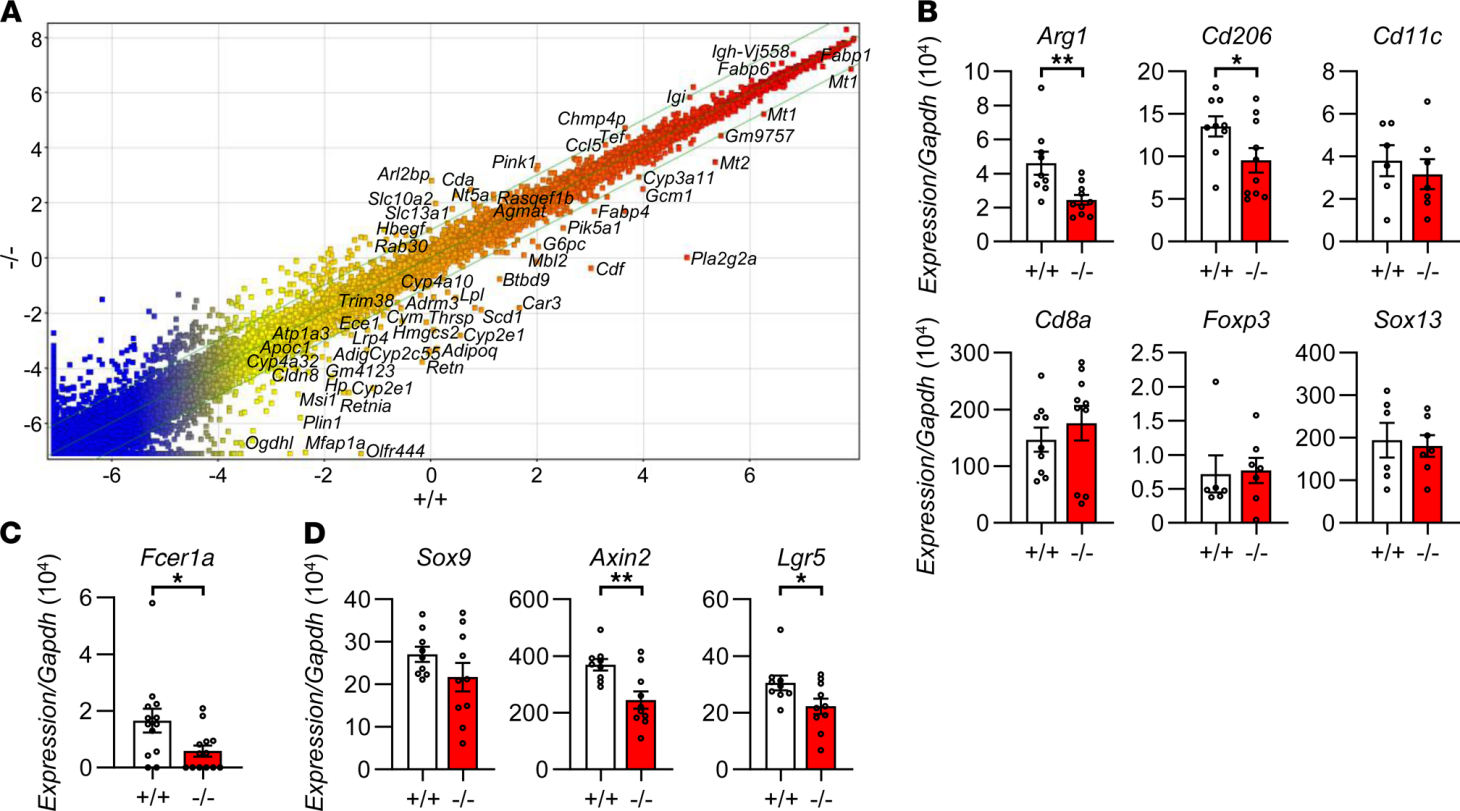
Altered gene expression profiles in the small intestine of *Pla2g2a*^–/–^ mice. (**A**) Scatter plot of gene expression profiles between *Pla2g2a*^+/+^ and *Pla2g2a*^–/–^ intestines. Samples from 4 mice were pooled for each genotype and then analyzed by microarray gene profiling. (**B**–**D**) qPCR of various immune cell markers (**B**), a mast cell marker (**C**), and intestinal Paneth cell or stem cell markers (**D**) (*n* = 6–13). Data are shown as mean ± SEM. *P < 0.05, **P < 0.01; Student *t* test. Data are compiled from 3 or 4 experiments (**B**–**D**).

To gain further insight into the role of intestinal sPLA_2_-IIA, we performed microarray gene profiling using the small intestines from *Pla2g2a*^−/−^ and *Pla2g2a*^+/+^ mice. A scatter plot of the microarray data revealed a notable alteration in the gene expression profile for *Pla2g2a*^–/–^ mice in comparison with *Pla2g2a*^+/+^ mice (Figure 4A). We noted that a panel of genes encoding the variable regions in the heavy and light chains of Ig were most dramatically altered in *Pla2g2a*^−/−^ mice relative to *Pla2g2a*^+/+^ mice (Supplemental Table 1A). This observation suggests that the antibody responses to commensal microbiota are distinct between the genotypes, lending additional support to the possibility that loss of intestinal sPLA_2_-IIA affects the gut microbiota. Furthermore, the expression levels of several genes related to immunity, the epithelial barrier, and lipid metabolism were also noticeably different between the genotypes (Supplemental Table 1B). The overall propensity implies that sPLA_2_-IIA deficiency allows the intestine to remain in a mildly proinflammatory state, even though the intestinal histology appeared to be normal in both genotypes. The highest increase of *Il12rb2* (IL-23 receptor β chain) in *Pla2g2a*^−/−^ mice relative to *Pla2g2a*^+/+^ mice might be a reflection of the increased Th17-type immunity (Supplemental Table 1B), which could be associated with psoriasis in distal skin (46). Gene ontology (GO) analysis (https://david.ncifcrf.gov/) revealed downregulation of several genes involved in PPAR signaling in *Pla2g2a*^–/–^ mice relative to *Pla2g2a*^+/+^ mice (Supplemental Table 1C), in agreement with the reduced expression of several PPARγ-dependent genes (e.g., *Adiq, Fabp4, Lpl, Scd1*, and *Plin1*) in the null mice (Supplemental Table 1B). Considering that PPAR signaling attenuates inflammation (47, 48), the reduced expression of a panel of genes in this signaling pathway might be related to the elevated expression of several inflammatory genes in *Pla2g2a^–/–^* mice (Supplemental Table 1B). Along with this, *Pla2g2a*^–/–^ mice had lower expression of 2 known markers for M2 macrophages (*Arg1* and *Cd206*), whose differentiation depends on PPAR signaling (49), although the expression of signature markers for other immune cells (*Cd11c, Cd8, Foxp3,* and *Sox13* for M1 macrophages and DCs, cytotoxic T cells, Tregs, and γδ T cells, respectively) was not profoundly affected by sPLA_2_-IIA deficiency (Figure 4B). Of note, the expression of *Fcera1a* (encoding FcεRI α chain, a mast cell marker) was lower in *Pla2g2a*^–/–^ mice than in *Pla2g2a*^+/+^ mice (Figure 4C), and this is indicative of disturbed mast cell differentiation and degranulation in *Pla2g2a*^–/–^ mice (Figure 1, I and J).

A recent study has shown that Tg overexpression of human *PLA2G2A* in C57BL/6 mice negatively regulates the differentiation of Paneth cells from intestinal stem cells through restriction of Yap-Wnt signaling (26). Our study, however, showed that expression of *Sox9* (a Paneth cell marker) was similar between *Pla2g2a*^+/+^ and *Pla2g2a*^–/–^ mice, while that of *Axin2* and *Lgr5* (Wnt signaling and stem cell markers, respectively) was significantly lower in *Pla2g2a*^–/–^ mice compared with *Pla2g2a*^+/+^ mice (Figure 4D). Thus, unlike the case in *PLA2G2A^TGN^* C57BL/6 mice (26), genetic deletion of sPLA_2_-IIA in BALB/c mice does not profoundly disturb Paneth cell differentiation, although it may have some impact on the stem cell niche, possibly reflecting a secondary effect of gut microbiota alteration.

### Altered blood metabolites in Pla2g2a^–/–^ mice.

**Figure 5:**
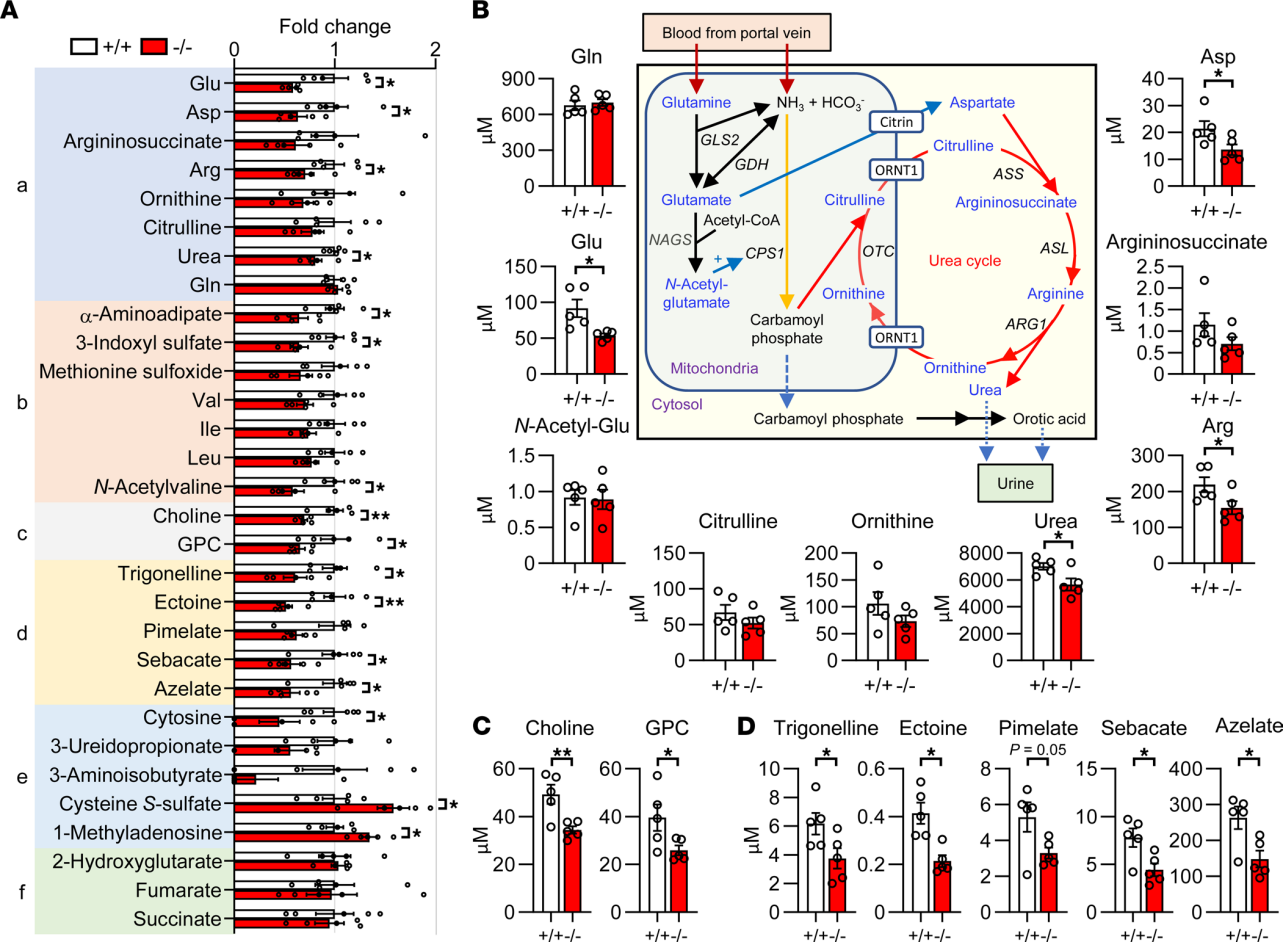
Metabolome analysis of hydrophilic metabolites in serum. (**A**) Relative abundance of various metabolites in the serum of *Pla2g2a*^–/–^ mice relative to *Pla2g2a*^+/+^ mice (*n* = 5). These metabolites are classified into those related to the urea cycle, ROS, choline metabolism, bacterial metabolites, others, and oncometabolites in the TCA cycle. (**B**) Quantification of metabolites in the urea cycle from **A**. The metabolic pathway in the urea cycle is illustrated. (**C**) Quantification of metabolites in choline metabolism from **A**. (**D**) Quantification of bacteria-specific metabolites from **A**. Data are shown as mean ± SEM. *P < 0.05, **P < 0.01; Student *t* test. Data are compiled from 2 experiments.

Since dysbiosis is accompanied by changes in microbial or host metabolites that affect host responses (2, 3), we performed comprehensive metabolome analysis of hydrophilic substances in the plasma of *Pla2g2a*^–/–^ and *Pla2g2a*^+/+^ mice. Of the 511 metabolites analyzed, approximately 160 were detected in plasma, among which 19 metabolites were significantly changed in *Pla2g2a*^–/–^ mice in comparison with *Pla2g2a*^+/+^ mice. These metabolites were categorized into several groups, including those related to (a) the urea cycle, (b) reactive oxygen species (ROS), (c) choline metabolism, (d) bacterial metabolites, (e) miscellaneous metabolites, and (f) oncometabolites in the TCA cycle (Figure 5A).

The urea cycle is associated with T cell immunity and tumor promotion (50, 51). Several metabolites in the urea cycle were concomitantly decreased in *Pla2g2a*^–/–^ mice relative to *Pla2g2a*^+/+^ mice (Figure 5B). This might be partly related to the reduced expression of *Arg1* (Figure 4B), which is responsible for the generation of urea from arginine (Figure 5B). *Pla2g2a*^–/–^ mice had lower levels of ROS-related metabolites, including α-aminoadipate, 3-indoxyl sulfate, methionine sulfoxide, and branched-chain amino acids (Supplemental Figure 4), which could be associated with altered systemic responses including inflammation and cancer (52, 53). Plasma levels of choline and glycerophosphocholine (GPC), whose aberrant accumulation is often associated with malignant transformation (54), were reduced in *Pla2g2a*^–/–^ mice (Figure 5C). In the intestinal tract, dietary and biliary phosphatidycholine (PC) are degraded to GPC and then to choline by sequential actions of phospholipases and phosphodiesterases. This raises the possibility that, in addition to sPLA_2_-IB, a pancreatic sPLA_2_ (55), sPLA_2_-IIA may act as another digestive sPLA_2_ to degrade dietary and biliary PC in the presence of detergent-like bile acids in the intestinal lumen. 

Some bacterial metabolites such as trigonelline and ectoine, which can modulate inflammation and cancer (56), and dicarboxylic acids such as pimelate, sebacate, and azelate, whose levels are associated with changes in gut microbiota such as *Ruminococcaceae* (57), were reduced in *Pla2g2a*^–/–^ mice relative to *Pla2g2a*^+/+^ mice (Figure 5D), providing additional insight into the view that sPLA_2_-IIA contributes to shaping of the intestinal microbiota. The decrease of cytosine, 3-ureidopropionate (a pyrimidine degradation product), and 3-aminoisobutyrate (a thymine degradation product) in *Pla2g2a*^–/–^ mice might be correlated with the decrease of their precursors aspartate and valine (Supplemental Figure 4). However, sPLA_2_-IIA deficiency did not affect the levels of 2-hydroxyglutarate, fumarate, and succinate (Figure 5A), ruling out the involvement of these TCA cycle–derived oncometabolites (58). Overall, sPLA_2_-IIA deficiency alters the blood levels of various metabolites that could affect carcinogenesis and inflammation.

### Altered fecal lipids in Pla2g2a^–/–^ mice.

**Figure 6:**
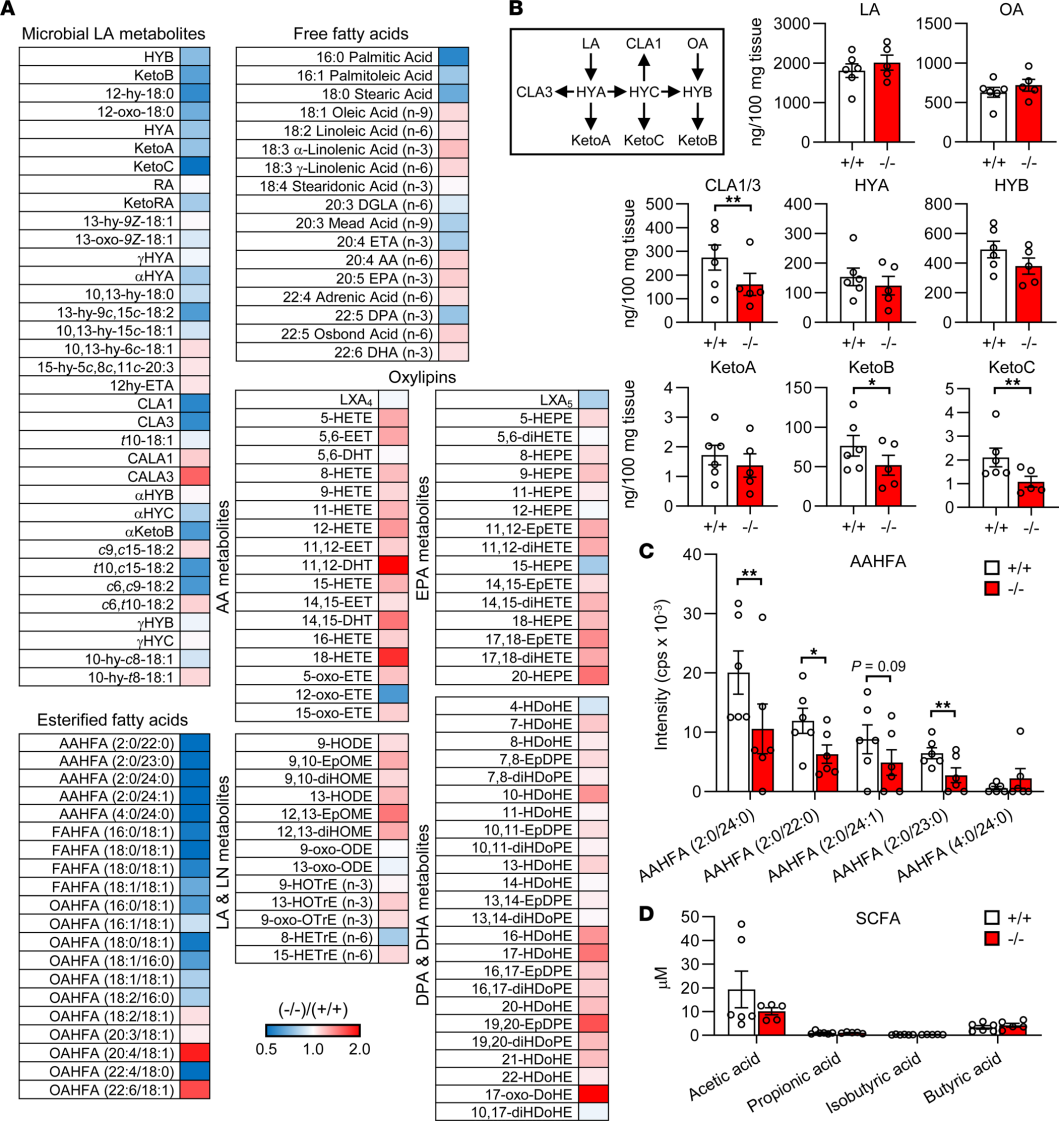
Lipidomics analysis of fecal lipids. (**A**) Heatmap representation of various lipids in the feces of *Pla2g2a*^–/–^ mice relative to *Pla2g2a*^+/+^ mice. These lipids are classified into microbial LA metabolites, esterified fatty acids, free fatty acids, and oxylipins derived from polyunsaturated fatty acids. (**B**–**D**) Quantification of microbial LA metabolites (**B**), esterified fatty acids (AAHFAs), and short-chain fatty acids (SCFAs) (**D**) (*n* = 5–6). Data are shown as mean ± SEM. *P < 0.05, **P < 0.01; Student *t* test. Data are representative of 2 experiments.

We then analyzed the stool lipid profiles of *Pla2g2a*^–/–^ mice relative to *Pla2g2a*^+/+^ mice by lipidomics analysis using liquid chromatography coupled with tandem mass spectrometry (LC-MS/MS). A heatmap representation of individual lipids (fatty acids and their oxygenated metabolites) is summarized in Figure 6A, and quantified data for representative lipids are shown in Figure 6, B–D.

A class of bacteria-specific metabolites derived from linoleic acid (LA; 18:2), such as 10-oxo-octadecanoic acid (KetoB), 10-oxo-trans-11-octadecenoicacid (KetoC), and conjugated LAs (CLA1/3; *cis*-9, *trans*-11- and *trans*-9, *trans*-11-octadecadienoic acids), was significantly reduced in *Pla2g2a*^–/–^ mice relative to *Pla2g2a*^+/+^ mice (Figure 6B). These bacterial LA metabolites have the capacity to attenuate inflammation and improve metabolism and barrier function (9, 59, 60). Another class of fatty acid metabolites that were mostly (even if not solely) decreased in *Pla2g2a*^–/–^ mice relative to *Pla2g2a*^+/+^ mice were branched fatty acid esters of hydroxy fatty acids (FAHFAs) including those having short-chain fatty acid esters (acyl α-hydroxy fatty acids; AAHFAs), as well as linear fatty acid esters (*O*-acyl-ω-hydroxy fatty acids; OAHFAs) (Figure 6A). Indeed, the levels of AAHFAs, in which a short-chain fatty acid (acetate, propionate, or butyrate) was esterified at the C-2 (α) position of a long-chain fatty acid (61, 62), were substantially lower in *Pla2g2a*^–/–^ mice than in *Pla2g2a*^+/+^ mice (Figure 6C). Long-chain FAHFAs exert antiinflammatory, antioxidant, and antidiabetic functions, possibly by acting on GPR40 or GPR120 (63, 64), and AAHFAs — likely produced by gut microbiota (62) — are inversely correlated with metabolic disease (61). Because OAFHA is a byproduct of the biosynthesis of the barrier lipid ω-*O*-acylceramide in keratinocytes (65), the decrease of several OAFHA species in the intestine of *Pla2g2a*^–/–^ mice might reflect perturbation of the gut epithelial barrier. Although short-chain fatty acids, which are produced by gut microbiota through fermentation of dietary fiber, have beneficial effects on metabolism and immunity by acting on GPR41, GPR43, or GPR109 or by inhibiting the histone deacetylase HDAC (66, 67), they did not differ significantly between the genotypes (Figure 6D).

In addition, various oxylipins (i.e., oxygenated metabolites of polyunsaturated fatty acids), including those derived from LA, linolenic acid (LN; 18:3), arachidonic acid (AA; 20:4), eicosapentaenoic acid (EPA; 20:5), docosapentaenoic acid (DPA; 22:5), and docosahexaenoic acid (DHA; C22:6), tended to be modestly more abundant in *Pla2g2a*^–/–^ mice than in *Pla2g2a*^+/+^ mice (Figure 6A and Supplemental Figure 5). Most of these metabolites were epoxy, hydroxy, and dihydroxy forms of polyunsaturated fatty acids, which can be produced by lipoxygenases or cytochrome P450s in both microbial and host cells and generally have antiinflammatory functions (68). In contrast, prostanoids, which are produced by host cyclooxygenases, were hardly detected in the feces, suggesting that the majority of these fecal lipid mediators are derived from the gut microbiota. Taken together, the data suggest that lack of sPLA_2_-IIA alters various fatty acid metabolites of bacterial origin in the gut, which could impact cancer and inflammation to various extents.

### Insights from Pla2g2a^–/–^ mice housed in the second animal facility.

While *Pla2g2a*^–/–^ mice and their littermates were initially housed in a specific pathogen–free (SPF) animal facility at the TMIMS, where all of the aforementioned analyses were carried out, the mice were subsequently moved into the second, more stringent SPF animal facility at the UTokyo, which had stricter access and personal protective equipment standards. Notably, intestinal *Pla2g2a* expression in WT mice housed in the UTokyo animal facility was lower than that in the first facility, being comparable with that seen in germ-free mice (Supplemental Figure 6A). Furthermore, in the UTokyo animal facility, IMQ-induced psoriasis was comparable between *Pla2g2a*^+/+^ and *Pla2g2a*^–/–^ mice (Supplemental Figure 6, B–D). In fact, ear swelling and psoriasis marker expression in both genotypes at the UTokyo facility were similar to those in single-housed *Pla2g2a*^–/–^ mice and cohoused *Pla2g2a*^+/+^ and *Pla2g2a*^–/–^ mice at the TMIMS facility (Supplemental Figure 6, C and D). These results indicate that the mice housed in the UTokyo facility might have lacked particular gut bacterial species that contribute to the upregulation of *Pla2g2a* expression, thus mitigating the effect of intestinal sPLA_2_-IIA on the distal skin.

OTU, cluster dendrogram, and PCoA analyses of gut bacterial 16S rRNA revealed that the abundance, diversity, and composition of gut microbiota were nearly identical between *Pla2g2a*^+/+^ and *Pla2g2a*^–/–^ mice housed in the UTokyo facility (Supplemental Figure 7, A–C). However, some species of *Ruminococcaceae* (*UGC-014* and *NK4A214* *group*) and *Lachnospiraceae* (*NK4B4 group* and *Marvinnryantia*), which were altered in *Pla2g2a^–/–^* mice at the TMIMS facility (Figure 3F), also showed a similar trend in those housed at the UTokyo facility (Supplemental Figure 7D), indicating that the sensitivity of these bacteria to sPLA_2_-IIA was conserved regardless of the different housing conditions. Notably, *Helicobacter* was undetectable in both *Pla2g2a*^+/+^ and *Pla2g2a*^–/–^ mice housed at the UTokyo facility, where the absence of *Helicobacter* is strictly controlled by a facility rule. In addition, *Ruminococcaceae* *UCG-013*, which was altered in *Pla2g2a*^–/–^ mice at the TMIMS facility (Figure 2H, Figure 3F, and Supplemental Figure 3), did not differ significantly between the genotypes at the UTokyo facility (Supplemental Figure 7D). Thus, the overall resemblance of gut microbiota, and possibly because of the absence of *Helicobacter* or no difference in *Ruminococcaceae* *UCG-013* or some other bacteria, at the UTokyo facility may render *Pla2g2a*^–/–^ mice less susceptible to the skin phenotypes.

## Discussion

Herein, using *Pla2g2a*^–/–^ BALB/c mice, a useful experimental tool for analyzing the specific role of sPLA_2_-IIA in the intestine, we have provided evidence that intestinal sPLA_2_-IIA serves as a modulator of the gut microbiota. Genetic deletion of *Pla2g2a* in BALB/c mice ameliorates skin carcinogenesis and conversely exacerbates psoriasis, despite its negligible expression in the skin. Since reduced skin cancer and increased psoriasis are also observed in mice lacking sPLA_2_-IID, which attenuates Th1/Th17 immunity by mobilizing ω3 fatty acids in lymphatic tissues (31), it is plausible that an altered systemic immunological balance may also underlie the skin phenotypes in *Pla2g2a*^–/–^ mice. Although the possibility that sPLA_2_-IIA expressed at very low levels in immune cells might also contribute, at least partly, to these phenotypes cannot be fully ruled out, the following lines of evidence support the idea that the skin phenotypes observed in *Pla2g2a*^–/–^ mice are largely attributable to an alteration of the microbiota in the intestine, the only tissue where sPLA_2_-IIA is robustly expressed. First, metagenome analysis of gut microbiota revealed an apparent difference in the fecal bacterial composition between *Pla2g2a*^+/+^ and *Pla2g2a*^–/–^ mice. Second, the skin phenotypes in *Pla2g2a*^–/–^ mice were lost after cohousing with *Pla2g2a*^+/+^ mice, and this allowed exchange of the gut microbiota through coprophagia. Third, likely in response to the change in gut microbiota, the expression profiles of a set of genes related to immunity, epithelial barrier function, and metabolism are altered in the intestine of *Pla2g2a*^–/–^ mice. Fourth, multiple microbial metabolites, which could influence immunity and cancer, are variably affected in the circulation and feces of *Pla2g2a*^–/–^ mice. Fifth, the reduced intestinal expression of *Pla2g2a* in the second animal facility, where *Pla2g2a*^+/+^ and *Pla2g2a*^–/–^ mice have a similar gut microbial composition, is associated with a disappearance of the skin phenotypes in *Pla2g2a*^–/–^ mice. Although we are no longer able to return *Pla2g2a*^–/–^ mice to the first animal facility and, thus, define the specific bacterial species and metabolites responsible for the skin phenotypes there, housing the mice in the second facility has provided another opportunity to gain further insight into the functional relationship between intestinal sPLA_2_-IIA and microbiota. Overall, we conclude that intestinal sPLA_2_-IIA plays a role in regulation of the gut microbiota and that its absence eventually leads to alterations in systemic host responses. To our knowledge, this is the first demonstration that a particular lipolytic enzyme secreted from intestinal Paneth cells controls the gut microbiota, thus opening a potentially new avenue in microbiome research.

The intestinal expression of *Pla2g2a* is markedly reduced by antibiotic treatment or housing in a germ-free facility, implying a feed-forward cycle of the sPLA_2_-IIA–microbiota interaction in that some microbial products upregulate the expression of sPLA_2_-IIA, which in turn modulates the microbial community and thereby impacts distal skin pathologies. In agreement, previous studies demonstrated that depletion of the microbiota in BALB/c mice by antibiotics reduced sPLA_2_-IIA expression by Paneth cells (69) and that the colonization of germ-free C3H mice with the microbiota of conventionally housed mice increased intestinal sPLA_2_-IIA expression (70). Indeed, LPS and peptidoglycan (components of Gram-negative and -positive bacteria, respectively) can activate NF-κB signaling, leading to sPLA_2_-IIA induction, although some virulence factors from Gram-positive bacteria counteract this process (19).

Although sPLA_2_-IIA kills Gram-positive bacteria more efficiently than Gram-negative bacteria in vitro (16, 20, 21), both Gram-positive (e.g., *Lachnospiraceae* and *Ruminococcaceae*) and Gram-negative (e.g., *Helicobacteraceae*) bacteria are affected by sPLA_2_-IIA deficiency in vivo, implying that the bactericidal activity of sPLA_2_-IIA could be modified by the copresence of antimicrobial factors in the gut lumen. This notion appears to be compatible with the action of lysozyme, a Paneth cell–derived bactericidal enzyme whose bacterial tropism in vitro is not entirely identical to that in vivo (7). However, the effect of sPLA_2_-IIA on gut microbiota could not be simply accounted for by only its bactericidal activity, since only specific bacteria were altered, while total microbial load and relative abundance of most bacteria were largely unaffected, by sPLA_2_-IIA deficiency. Moreover, several genera of Gram-positive *Lachnospiraceae* and *Ruminococcaceae* were decreased, rather than increased, in *Pla2g2a*^–/–^ mice. These results suggest that the altered gut microbiota in *Pla2g2a*^–/–^ mice may also involve complex interactions between different bacterial species and between bacteria and host. Conceivably, sPLA_2_-IIA may increase certain bacteria through eradicating competing commensals, a view that is reminiscent of the situation in patients with cystic fibrosis, where sPLA_2_-IIA–resistant *Pseudomonas aeruginosa* upregulates the expression of sPLA_2_-IIA, which then eradicates the sPLA_2_-IIA–sensitive *Staphylococcus aureus*, allowing the former bacterium to become dominant within the lung niche (22). Other possibilities include indirect actions of sPLA_2_-IIA via stimulation of the host immune system involving the production of lipid mediators from host cells or extracellular vesicles (16–18) and via the sPLA_2_-receptor–dependent (PLA2R1-dependent) mechanism (26). Although our lipidome and transcriptome analyses do not support a profound contribution of these mechanisms, it is still possible that certain lipid mediators and/or PLA2R1, along with direct bacterial membrane hydrolysis, might underlie the sPLA_2_-IIA action.

Among various microbial species detected so far, *Helicobacter* is one of those most profoundly affected by sPLA_2_-IIA deficiency, being enriched in *Pla2g2a*^–/–^ mice relative to *Pla2g2a*^+/+^ mice. This finding is complementary to a study demonstrating that *H*. *felis* infection is prevented in *PLA2G2A^TGN^* mice (43). Moreover, gut expression of sPLA_2_-IIA in BALB/c mice is upregulated by *H*. *felis* infection, and mouse strains that intrinsically lack sPLA_2_-IIA (e.g., C57BL/6 and 129/Sv) are more susceptible to *Helicobacter*-induced enteritis than strains expressing sPLA_2_-IIA (e.g., BALB/c and C3H) (71), further implying the functional relationship between *Helicobacter* and sPLA_2_-IIA. *Helicobacter* infection is generally known to be associated with gastric cancer, as well as colon and liver cancers (40, 41), while several studies have pointed out that *Helicobacter* has an opposite, beneficial impact on cutaneous responses (44, 45). Importantly, the increased presence of *Helicobacter* in *Pla2g2a*^–/–^ mice could explain why mouse strains with a mutated *Pla2g2a* gene are more prone to intestinal tumorigenesis than those with a native *Pla2g2a* gene (28), why Tg transfer of the *Pla2g2a* gene into *Pla2g2a*-null C57BL/6 mice reduces the incidence of intestinal polyposis (72), and why *PLA2G2A* expression is inversely correlated with gastric cancer in humans (73). Thus, the effect of sPLA_2_-IIA on *Helicobacter* might be one of the key determinants critically affecting susceptibility to cancer, psoriasis, and possibly other disorders in proximal and distal tissues with different outcomes. The absence of skin phenotypes in *Pla2g2a*^–/–^ mice housed in the *Helicobacter*-free UTokyo animal facility supports this hypothesis. Nevertheless, we do not fully rule out the possibility that some other bacteria species might additionally or distinctly contribute to the skin phenotypes or that the altered host immunity might eventually influence the gut microbiota in *Pla2g2a*^–/–^ mice.

Microarray gene profiling demonstrated that loss of sPLA_2_-IIA leads to modest but noticeable alterations in intestinal gene expression profiles, with a trend toward increased expression of proinflammatory genes and decreased expression of antiinflammatory genes, suggesting that the intestine in *Pla2g2a*^–/–^ mice has low-grade inflammation. This event could be at least partly explained by the increased presence of the proinflammatory bacterium *Helicobacter*, which contributes to exacerbation of enteritis (see above), and the decreased presence of the antiinflammatory bacterium *Ruminococcaceae*, which prevents colitis and promotes tissue repair (7), in *Pla2g2a*^–/–^ mice. Moreover, the altered expression of Ig genes may mirror the change in gut microbiota, and lower FcεRI expression may be related to the perturbed mast cell differentiation and may thereby reduce the presence of skin cancer in *Pla2g2a*^–/–^ mice. Thus, the sustained alteration in gut immune responses caused by sPLA_2_-IIA ablation may exert secondary systemic effects, leading to skin phenotypes. In fact, our results appear in line with the finding that increased intestinal inflammation could be associated with exacerbation of psoriasis (46) and the finding that there is a relationship between *Helicobacter* infection and psoriasis severity (74).

Metabolome analysis revealed that sPLA_2_-IIA deficiency alters the levels of various water-soluble metabolites in plasma, such as those related to the urea cycle, ROS generation, and choline metabolism — many of which have been implicated in inflammation and cancer (50, 51, 52, 54). Also, several classes of fecal fatty acid metabolites with antiinflammatory potential, which seem to be derived from the gut microbiota rather than from the host, are altered in *Pla2g2a*^–/–^ mice. These changes include a reduction of bacterial LA metabolites and FAHFAs, as well as modest increases of various oxylipins that are likely derived from polyunsaturated fatty acids through the action of microbial lipoxygenases or cytochrome P450s, in *Pla2g2a*^–/–^ mice (9, 63, 64). Because of the alterations in various hydrophilic and hydrophobic metabolites revealed thus far, it is difficult at this stage to determine any key metabolites that would truly affect the skin phenotypes in *Pla2g2a*^–/–^ mice. We speculate that the cooperative action of such multiple metabolites on immunity and metabolism might underlie the alterations of skin phenotypes resulting from sPLA_2_-IIA deficiency.

In the accompanying paper (75), Doré et al. have found that *PLA2G2A^TGN^* mice on a C57BL/6 genetic background exhibited age-associated systemic inflammation with lymphomegaly, granulocytosis, elevation of circulating IL-17A, and increased arthritis, due to alteration of the gut microbiota and bacteria-derived lipids. As in the case of *Pla2g2a*^–/–^ mice shown here, the phenotypes in *PLA2G2A^TGN^* mice are less severe after treatment with antibiotics or after housing in a more stringent SPF facility and can be modified by fecal transfer (cohousing in our study) through oral gavage. Although the results obtained from these 2 studies are not necessarily complementary, as they were performed using different disease models in KO versus Tg mice on different genetic backgrounds (BALB/c versus C57BL/6, respectively) using different approaches and housing conditions (e.g., animal facilities, food, water, and countries), both studies have reached the same conclusion that sPLA_2_-IIA acts as a host factor that is primarily expressed in the intestine and contributes to shaping of the gut microbiota, whose perturbation by genetic deletion or overexpression culminates in systemic effects, thus opening a potentially new avenue for the action modes of the sPLA_2_ family.

Given that several sPLA_2_ isoforms are expressed in the gut epithelium (76, 77), it is tempting to speculate that they may play a general role as a class of gut microbiome regulators. Indeed, genetic deletion of sPLA_2_-IB, a digestive sPLA_2_ that is secreted from the pancreas into the intestinal lumen to degrade dietary and biliary phospholipids (55), also alters gut microbiota (78), although it remains unclear whether or not this event would be functionally linked to the amelioration of obesity-related phenotypes in *Pla2g1b*^–/–^ mice (79, 80). sPLA_2_-X, which is abundantly expressed in the colonic epithelium (76, 81), might also contribute to the shaping of the gut microbiota, which might help to explain why the inflammatory, cardiovascular, and metabolic phenotypes in *Pla2g10*^–/–^ mice reported so far are not necessarily consistent among different laboratories (81–83). The functional interaction of other sPLA_2_s with gut microbiota and its impacts on systemic responses are now under investigation.

Lastly, considering that sPLA_2_-IIA is expressed in human skin (36, 84), translation of our present study to human skin pathology is not so simple, since we need to think of the skin-intrinsic roles of sPLA_2_-IIA and the extrinsic effects of the intestinal sPLA_2_-IIA/microbiota axis, both of which can be variably influenced by environmental factors. Nevertheless, in the context of skin cancer, our results have raised the possibility that the elevated levels of sPLA_2_-IIA in human feces might have predictive values for the disease. Moreover, the use of sPLA_2_ inhibitors or neutralizing antibodies might represent a potential therapy to prevent or treat skin cancer.

## Methods

### Mice.

*Pla2g2a^–/–^* BALB/c mice were generated by crossing BALB/c (*Pla2g2a*-sufficient) and C57BL/6 (*Pla2g2a*-mutated) mice, as previously described (30). Litters were selected for the heterozygous expression of the mutant *Pla2g2a* gene and backcrossed with BALB/c for 10 generation. For genotyping, the following set of primers were used: *Pla2g2a*-forward (common to both WT and mutant) 5′-CAGAGCTGACAGCATGAAGGTCCTC-3′; *Pla2g2a*^+/+^-reverse 5′-TCTGTGGCATCCTTGGGGGAT-3′; and *Pla2g2a*^–/–^-reverse 5′-CTGTGGCATCCTTGGGGGAA-3′. WT and mutant *Pla2g2a* gene fragments were amplified in 2 separate PCR conditions, and thermal cycles were set to 2 minutes at 95°C followed by 35 cycles comprising a denaturation step (30 seconds at 95°C) and an annealing and extension step (30 seconds at 67°C for WT or 64°C for the mutant, followed by 70°C for 60 seconds). Mice were maintained in the TMIMS and UTokyo animal facilities under SPF conditions. Heterozygous male and female *Pla2g2a*^+/–^ mice were intercrossed to obtain homozygous *Pla2g2a*^–/–^ mice and littermate *Pla2g2a*^+/+^ mice. Germ-free BALB/c mice were purchased from Japan SLC. All animal models were carried out with 8- to 12-week-old male mice.

### Skin carcinogenesis.

The back skin of mice was shaved with an electric clipper, and then 200 μL of 2 mM DMBA (Sigma-Aldrich) in acetone (31, 32) was applied. After 1 week, 200 μL of 80 μM TPA (Sigma-Aldrich) in acetone was applied to the skin twice a week over 24 weeks. Cutaneous papillomas were counted and scored weekly. The skin from the mice was subjected to histochemistry and qPCR.

### Psoriasis.

Mice received a daily topical application of 12.5 μg of 5% (w/v) IMQ (Mochida Pharma) on the dorsal and ventral surfaces of the ears for 6 days (75 μg of IMQ cream per mouse) (31, 32). Ear thickness was monitored with a micrometer. On day 6, the ear skin was subjected to qPCR.

### Histological analysis.

Tissues from mice were fixed with 4% paraformaldehyde and embedded in paraffin, and 5 μm sections were cut and stained with H&E or toluidine blue.

### Flow cytometry.

Mouse skin was incubated with 0.25% (w/v) trypsin-EDTA (Thermo Fisher Scientific) for 60 minutes at 37°C to separate the dermis from the epidermis. These tissues were then incubated with 400 units/mL collagenase type II (Worthington) with shaking for 30 minutes at 37°C. After adding 10 mM EDTA, the resulting cell suspensions were passed through a 70 μm cell strainer (Corning), and RBCs were then lysed for 2 minutes on iced with 10 mM Tris-HCl (pH 7.0) containing 0.84% (w/v) ammonium chloride. The cells were preincubated with purified rat anti–mouse CD16/CD32 (mouse BD Fc Block, clone 2.4G2, BD Biosciences) and then stained with fluorochrome-conjugated monoclonal antibodies specific for mouse F4/80 (clone BM8), CD11c (clone N418), and CD206 (clone C068C2), purchased from BioLegend. Flow cytometry was performed on a EC800 Cell Analyser flow cytometer (Sony Biotechnology) and analyzed using FlowJo software.

### Antibiotics treatment.

Mice were orally administered an antibiotic cocktail containing ampicillin (1 g/L; Nakarai Tesque), vancomycin (500 mg/L; Nakarai Tesque), neomycin sulfate (1 g/L; Sigma-Aldrich), and metronidazole (1 g/L; Sigma-Aldrich) in drinking water (85).

### 16S rRNA–based metagenome analysis.

Microbial DNA was extracted from fecal samples by the bead beating method with a slight modification (86). Fecal samples were mixed with 0.5 mL of lysis buffer (no. 10, Kurabo Industries) and 0.5 g of 0.1 mm glass beads and then homogenized using a PS1000 cell destroyer (Bio Medical Science) at 4260 rpm for 50 seconds at room temperature. The homogenized sample was centrifuged (12,000*g* for 5 minutes at room temperature), and the supernatant was collected and mixed with lysis buffer (No. 10, Kurabo Industries) and proteinase K buffer (no. 2, Kurabo Industries) containing proteinase K. DNA was then extracted using a Gene Prep Star PI-80X device (Kurabo Industries). The concentration of the extracted DNA was determined using a NanoDrop Spectophotometer ND-1000 (Thermo Fisher Scientific).

For 16S rRNA gene amplification and sequencing, the V3–V4 region of the 16S rRNA was amplified from fecal DNA samples using primers specific for the V3–V4 regions (87). A DNA library was prepared using Nextera kit set A (Illumina), and 16S rRNA gene sequencing was performed using Illumina MiSeq (Illumina) in accordance with the manufacturer’s instructions, producing a total of ~225,000 reads per sample; 10,000 reads per sample were randomly selected for further analysis. Samples with insufficient read numbers were resequenced, and any remaining samples with insufficient read numbers were excluded thereafter. The obtained paired end FASTQ data were trimmed and merged before selection of the OTUs. The OTU classification and diversity analysis were performed using the QIIME pipeline (v1.9.1) (88). All the steps from FASTQ trimming to gut microbiota diversity analysis were performed automatically using a previously described method (89). The OTUs were clustered against the SILVA 128 reference database (90) at 97% similarity using the USEARCH algorithm (91). Taxonomic classification to the genus level was performed using the SILVA 128 reference database. Specific taxonomic names were expressed on the basis of the SILVA database phylogenetic classification standard (https://www.arb-silva.de/browser/ssu/).

The output of QIIME pipeline in Biom table format was imported and analyzed in R (version 3.5.1). The α-diversity indices were calculated by the estimate richness function in the “phyloseq” R-package. Hierarchical clustering analysis was performed using the R package “vegan”, “stats” based on the Bray-Curtis distance matrix at the genus level and by using the ward.D2 method. Random forest classification analysis was performed to confirm the potential contribution of gut microbiota between *Pla2g2a*^+/+^ and *Pla2g2a*^–/–^ mice. The data set of the classification random forest prediction model was evaluated using all data as training data. In the data set, descriptors that showed near-zero variance was identified and excluded by calculating the frequency ratio using the *nearZeroVar* function in the “caret” package. Thereafter, descriptors that significantly contributed to the prediction accuracy were selected using the Boruta algorithm to automatically rank and omit descriptors based on the random forest classification algorithm with the training set. Random forest classification analysis was performed using the train function of the “caret” R package.

### Microarray.

Total RNA extracted from mouse small intestine was purified using the RNeasy Mini Kit (Qiagen). The quality of the RNA was assessed using a 2100 Bioanalyzer (Agilent Technologies). Gene expression profiling was performed using the Agilent Technologies profiling system. Cy3-labeled cRNA targets were amplified using a one-color Low Input Quick Amp Labeling Kit and hybridized with a Whole Mouse Genome Gene Expression 4x44K Microarray in accordance with the manufacturer’s instructions. Arrays were scanned on an Agilent Microarray Scanner to detect fluorescence. For data processing, the scanned images were analyzed using Feature Extraction software. The microarray data were analyzed using GeneSpring GX software. The GEO accession no. for the microarray is GSE182283.

### qPCR analysis.

The reagents and instrument required for qPCR were purchased from Thermo Fisher Scientific. Total RNA from mouse tissues was isolated using TRIzol reagent in accordance with the manufacturer’s instructions. First-strand cDNA synthesis was performed using a High Capacity cDNA Reverse Transcription Kit. qPCR was performed with a predesigned primer-probe set (TaqMan Gene Expression Assay) and TaqMan Gene Expression Master Mix on a StepOnePlus Real-Time PCR System (Supplemental Table 2).

### Metabolome analysis.

Metabolome analysis was performed at Human Metabolome Technologies (HMT), as described previously (92). Briefly, frozen mouse blood samples were homogenized in extraction solvent containing the internal standards. The metabolites were extracted in accordance with the provided protocol (93). The samples were subjected to capillary electrophoresis–TOFMS (CE-TOFMS) analysis using the Agilent CE-TOFMS System (Agilent Technologies) for global analysis of charged metabolites. The metabolites were identified by comparing their *m/z* values and relative migration times with those of metabolite standards. Quantification was performed by comparing the peak areas with calibration curves generated using internal standardization techniques. CE-TOFMS raw data were analyzed using MasterHands software.

### Lipidomics analysis.

Fecal lipidomic profiling was performed at the Laboratory for Metabolomics, RIKEN Center for Integrative Medical Sciences, as described previously (62). Briefly, collected mouse feces were homogenized in methanol, and lipids were obtained by single-phase extraction. Lipids were separated on an Acquity UPLC Peptide BEH C18 column (Waters). Oxidized fatty acids, including LA metabolites, were extracted using a Sep-Pak C18 cartridge (Waters) and analyzed by LC-quadrupole MS (QTRAP4500 or QTRAP5500; Sciex). Untargeted lipidomics was performed using an ACUITY UPLC system (Waters) coupled with QTOF-MS (TripleTOF 5600^+^ or TripleTOF 6600; Sciex) (93).

### Statistics.

Results are presented as means ± SEM or median ± IQR. Statistical significance was evaluated by 2-tailed Student *t* test, Mann-Whitney *U* test, 1-way ANOVA with Tukey’s or Dunnett’s multiple comparison tests, and Kruskal-Wallis test using Prism (GraphPad software). Differences at *P* < 0.05 were considered to be significant.

### Study approval.

All animal experiments were approved by the IACUC of the TMIMS (approval nos. 13045, 14060, 15801, 16074, 17005) and the UTokyo (approval nos. 17-P-032, 20-P-104), and they conformed to the Japanese Guide for the Care and Use of Laboratory Animals.

## Author contributions

MM, YM, YT, and YK designed the study and wrote the manuscript. YN performed the microarray analysis. JP, KH, K Mizuguchi, and JK conducted the microbiome analysis. TS carried out the metabolome analysis of hydrophilic substances. SGBG, KI, and MA performed the lipidomics analysis. EB generated *Pla2g2a*^–/–^ mice. KY and K Muramatsu assisted with several parts of the experiments and data analysis.

## Supplementary Material

Supplemental data

## Version 1 01/25/2022

Electronic publication
