# Supplementary material for: Group IIA secreted phospholipase A2 controls skin carcinogenesis and psoriasis by shaping the gut microbiota
Source: JCI Insight. 2022 Jan 25;7(2):e152611. doi: 10.1172/jci.insight.152611 (PMC8855835; doi:10.1172/jci.insight.152611)
Supplement: Supplemental data [file jciinsight-7-152611-s088.pdf]

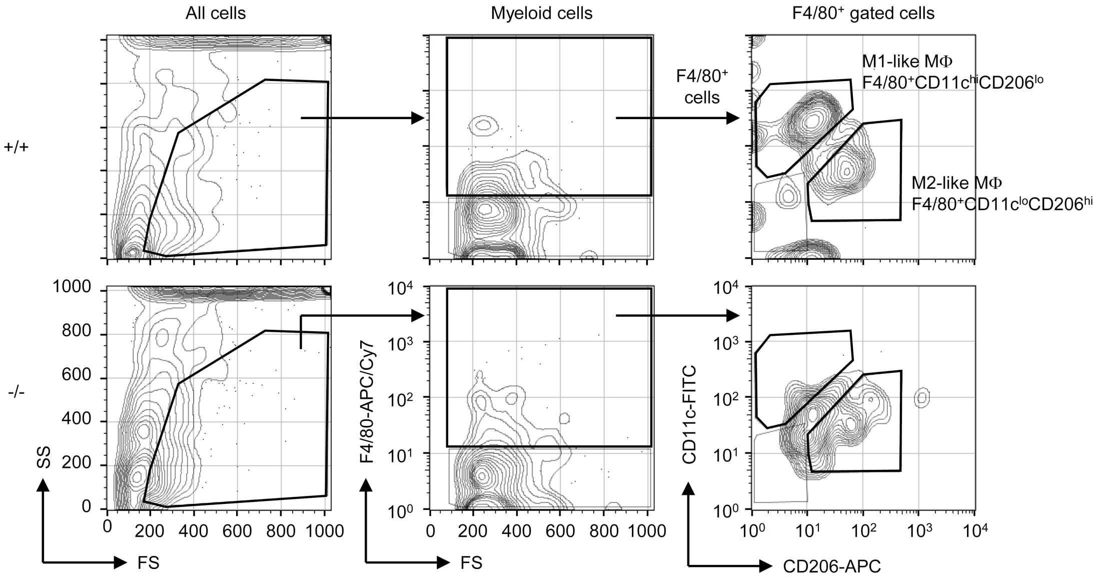

Supplemental Figure 1

Flow cytometry of macrophages in the skin of *Pla2g2a*$^{+/+}$ and *Pla2g2a*$^{-/-}$ mice.

Flow cytometric profile of M1- and M2-like macrophages in the skin of *Pla2g2a*$^{+/+}$ and *Pla2g2a*$^{-/-}$ mice at 24 weeks (related to Fig. 1G). Representative results of 3-4 mice for each genotype are shown.

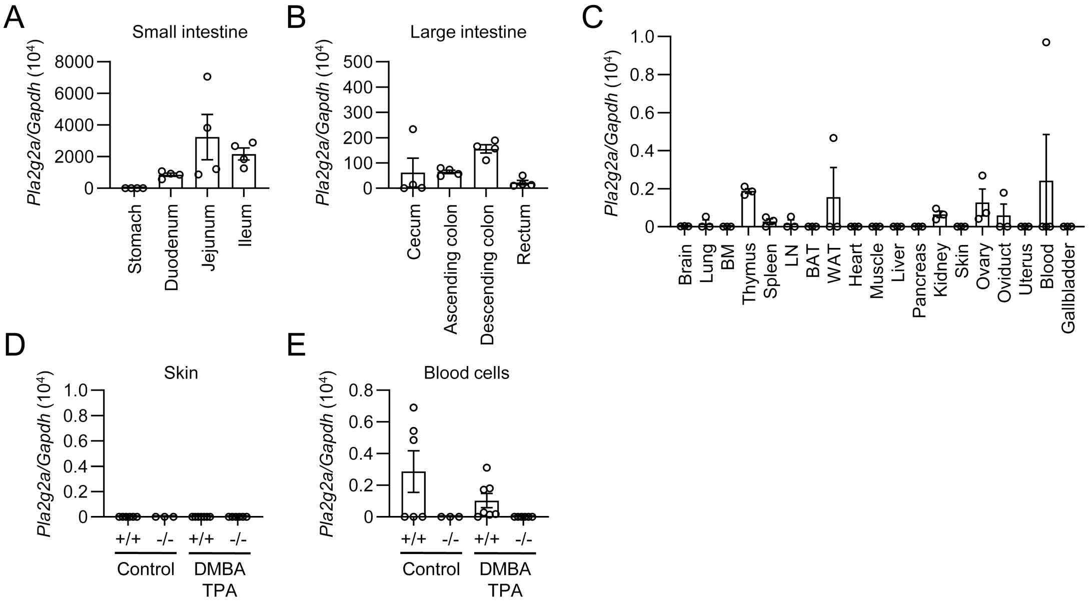

Supplemental Figure 2

Expression of *Pla2g2a* in various tissues of BALB/c mice.

(A-C) Quantitative RT-PCR of *Pla2g2a* in the small (A) and large (B) intestines as well as in other tissues (C) (n = 3-4). (D, E) Quantitative RT-PCR of *Pla2g2a* in the skin (D) and blood cells (E) with or without DMBA/TPA treatment for 24 weeks (n = 3-7). Values are mean ± s.e.m. Results are from one experiment.

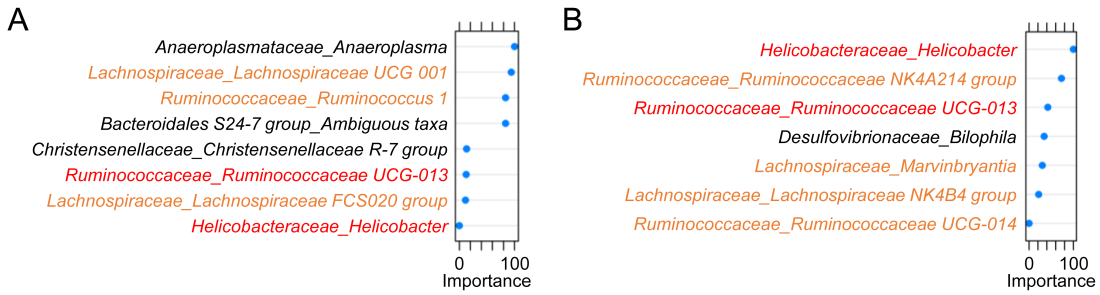

Supplemental Figure 3

Random forest analysis of the fecal microbiota between *Pla2g2a*$^{+/+}$ and *Pla2g2a*$^{-/-}$ mice.

The gut microbiota signatures, which resulted from the random forest classification analysis of *Pla2g2a*$^{+/+}$ (WT) and *Pla2g2a*$^{-/-}$ (KO) mice in two independent experiments (A and B), are shown. AUC = 1.000 (n = 6 for WT and n = 5 for KO) and 0.994 (n = 7 for each genotype) in (A) and (B), respectively. Bacteria belonging to the *Helicobacteraceae*, *Ruminococcaceae* and *Lachnospiraceae* families were commonly affected (orange), among which two particular bacteria, *Helicobacter* and *Ruminococcaceae UCG-013*, were reproducibly detected (red), in both experiments.

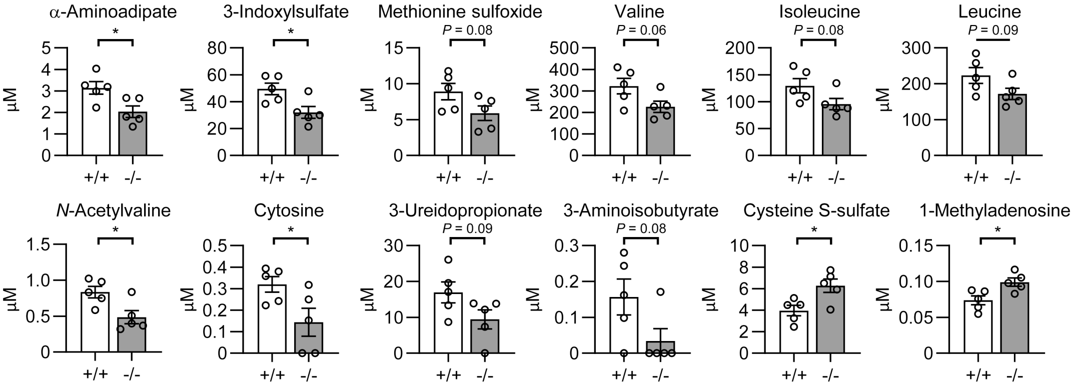

Supplemental Figure 4

Metabolome analysis of hydrophilic metabolites in serum.

Quantification of various metabolites in the serum of *Pla2g2a*$^{-/-}$ mice relative to *Pla2g2a*$^{+/+}$ mice (n = 5) (related to (b) and (e) in Figure 5A). A representative result of two experiments is shown. Values are mean ± s.e.m., **P* < 0.05, ***P* < 0.01; Student t test. Data are compiled from two experiments.

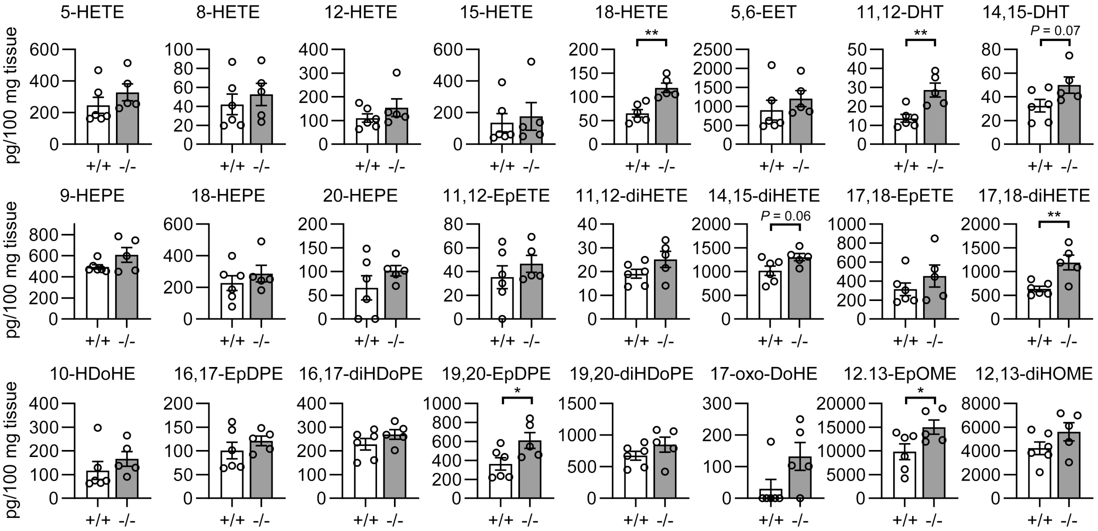

Supplemental Figure 5

Lipidomics analysis of oxylipins in feces.

Quantification of oxylipins in feces of *Pla2g2a*$^{+/+}$ and *Pla2g2a*$^{-/-}$ mice (n = 5-6) (related to Figure 6A). Values are mean ± s.e.m., *P < 0.05, **P < 0.01; Student t test. Data are representative of two experiments.

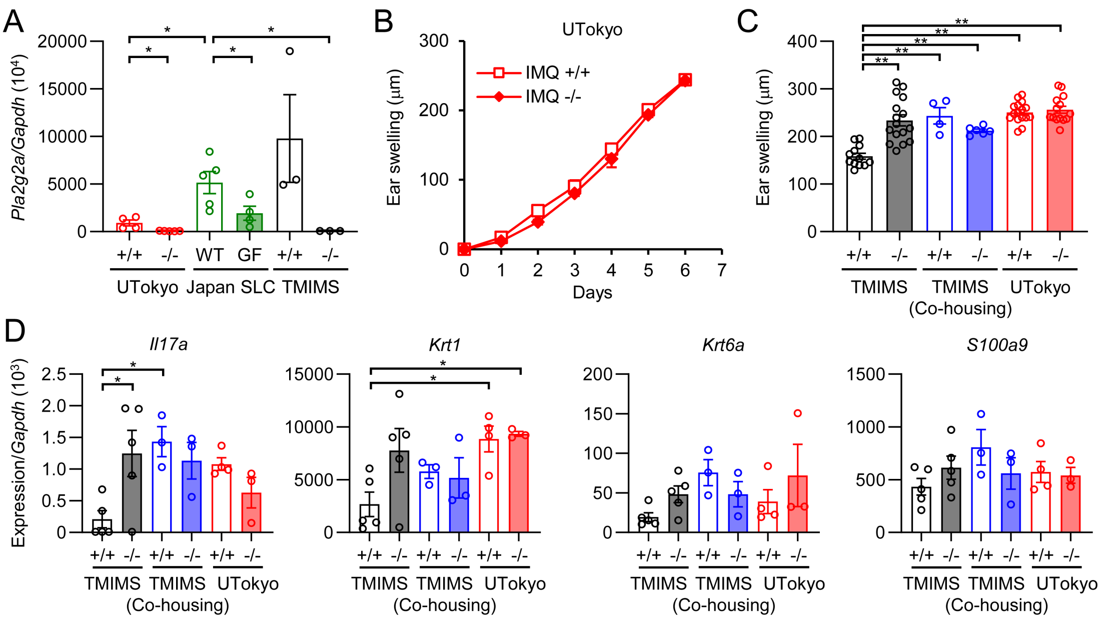

Supplemental Figure 6

Distinct psoriasis responses in *Pla2g2a*$^{-/-}$ mice under different housing conditions.

(A) Quantitative RT-PCR of *Pla2g2a* in the small intestine of *Pla2g2a*$^{+/+}$ and *Pla2g2a*$^{-/-}$ mice maintained in different facilities (UTokyo (n = 4-5) and TMIMS (n = 3)) as well as those obtained from Japan SLC (n = 4-5). Values are mean ± s.e.m. *$P < 0.05$, **$P < 0.01$; one-way ANOVA. (B) Time course of ear swelling in IMQ-treated *Pla2g2a*$^{+/+}$ and *Pla2g2a*$^{-/-}$ mice housed at the UTokyo animal facility (n = 8). (C, D) Comparison of IMQ-induced ear swelling (C) and expression of several psoriasis markers (D) in *Pla2g2a*$^{+/+}$ and *Pla2g2a*$^{-/-}$ mice housed at different facilities with or without co-housing on day 6 (n = 3-16). Values are mean ± s.e.m. *$P < 0.05$, **$P < 0.01$ *versus* *Pla2g2a*$^{+/+}$ mice housed at the TMIMS animal facility without co-housing; one-way ANOVA. Data are compiled from two experiments (A, C) or representative of two experiments (B, D).

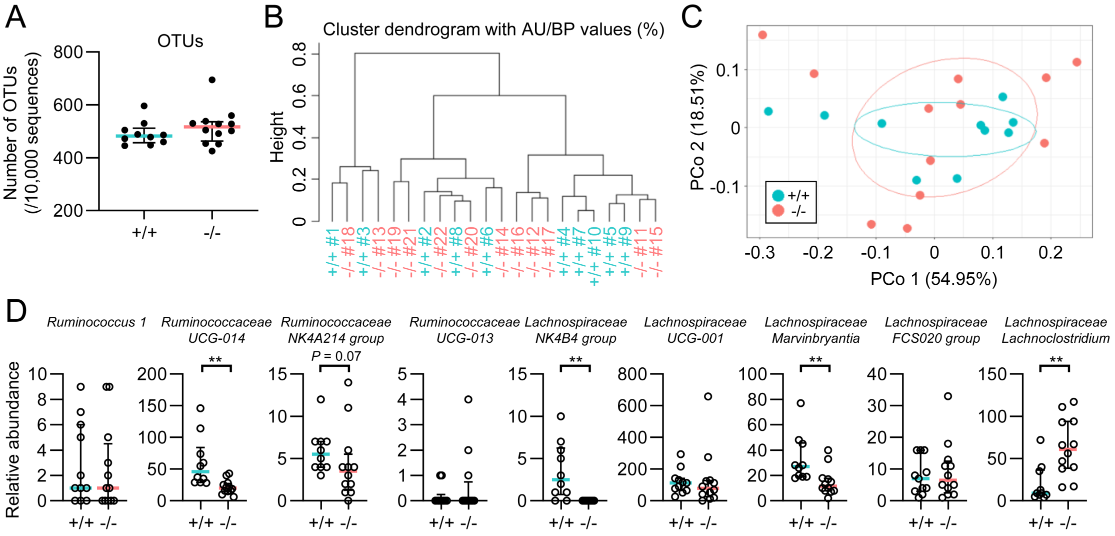

Supplemental Figure 7

Analysis of gut microbiota in *Pla2g2a*$^{+/+}$ and *Pla2g2a*$^{-/-}$ mice housed in a different animal facility.

(A-C) Shannon bacterial diversity (A), hierarchical clustering with β-diversity (B), and PCoA analysis based on unifrac phylogenetic distances (C) of fecal microbiota in *Pla2g2a*$^{+/+}$ mice (n = 10) and *Pla2g2a*$^{-/-}$ mice (n = 12) housed at the UTokyo animal facility. (D) Relative abundance of specific bacteria species in feces of *Pla2g2a*$^{+/+}$ and *Pla2g2a*$^{-/-}$ mice housed at the UTokyo animal facility. Values indicate median ± i.q.r. ***P* <0.01; Mann-Whitney U test. Data are compiled from two experiments.

Supplemental Table 1

Microarray gene profiling of the small intestine of *Pla2g2a*$^{-/-}$ mice relative to *Pla2g2a*$^{+/+}$ mice. Samples from four mice were pooled for each genotype and then analyzed by microarray gene profiling. Representative genes that were increased (red) or decreased (blue) in *Pla2g2a*$^{-/-}$ mice relative to *Pla2g2a*$^{+/+}$ mice. These genes included those related to immunoglobulins (A) as well as those related to immunity, epithelial barrier function, and lipid metabolism (B). (C) Ontology analysis of the genes that were decreased in *Pla2g2a*$^{-/-}$ mice in comparison with *Pla2g2a*$^{+/+}$ mice.

A

Probe name	[-/-]/[+/+]	Signal [+/+]	Signal [-/-]	Gene symbol
A_55_P1959421	25.70	18.86	486.50	*Igkv6-29*
A_55_P2075393	10.70	50.93	546.80	*Igkv6-20*
A_52_P84800	7.31	64.44	472.97	*Igkv13-84*
A_55_P2133907	6.32	62.96	399.21	*Ighv8-5*
A_52_P538084	5.46	196.03	1074.35	*Igkv1-135*
A_66_P119964	5.37	28.67	154.40	*Ighv1-78*
A_51_P115601	5.35	236.92	1272.65	*Igkv5-48*
A_66_P127563	5.32	120.30	641.87	*Igkv4-54*
A_66_P103124	5.18	24.39	126.68	*Ighv9-4*
A_55_P2140286	5.14	26.08	134.46	*Igkv1-131*
A_55_P2047974	4.89	157.57	773.48	*Ighv1-4*
A_55_P2164102	4.82	16.27	78.74	*Ighv1-12*
A_55_P2067620	4.78	28.89	138.66	*Igkv2-137*
A_52_P443776	4.77	49.63	237.45	*Ighv1-58*
A_55_P2187235	4.69	99.05	465.77	*Ighv2-2*
A_55_P2117342	4.63	371.76	1726.20	*Igkv2-112*
A_55_P1999033	4.57	46.06	211.48	*Ighv8-12*
A_55_P2065506	4.56	110.81	507.56	*Ighv1-36*
A_66_P105596	4.50	213.06	961.83	*Igkv3-12*
A_55_P1967286	4.37	102.19	448.54	*Igkv5-39*
A_66_P123324	4.33	11.81	51.26	*Igkv14-100*
A_52_P614207	4.32	361.25	1564.52	*Igkv3-7*
A_55_P2187234	4.29	63.85	274.67	*Ighv2-2*
A_55_P1988994	4.27	509.05	2183.19	*Igkv1-122*
A_55_P2167347	4.25	22.37	95.41	*Igkv14-126*
A_55_P1977451	4.24	553.30	2355.53	*Igkv4-80*
A_52_P630429	4.12	329.40	1361.45	*Igkv2-112*
A_52_P265556	-10.49	1487.86	142.34	*Igkv4-91*
A_55_P1997648	-12.52	648.70	52.00	*Ighv5-17*

B

	Probe name	[-/-]/[+/+]	Signal +/+	Signal -/-	Gene symbol
Immunity	A_55_P1978416	12.00	10.00	120.44	*Il12rb2*
	A_52_P578732	2.80	39.68	111.46	*Ccr5*
	A_55_P1992592	2.70	63.43	172.04	*Il5ra*
	A_51_P169476	2.69	280.95	757.31	*Mcpt1*
	A_51_P464703	2.69	353.95	956.99	*Ccl8*
	A_55_P2054315	2.65	211.04	560.42	*Mcpt2*
	A_55_P2050872	2.28	36.40	83.42	*Fcrlb*
	A_55_P1984556	2.16	76.97	166.74	*Ccl12*
	A_51_P145132	2.07	849.67	1761.28	*Mcpt4*
	A_55_P2141395	2.04	366.81	749.58	*Ly6g6c*
	A_55_P2010429	-6.50	81.33	12.56	*Tcrg-V4*
Epithelial barrier	A_55_P1974377	3.24	47.43	154.18	*Defb26*
	A_55_P2115906	3.14	69.49	218.86	*Dsg1c*
	A_52_P549190	-2.24	274.80	122.85	*Cldn8*
	A_55_P2112459	-2.53	62.87	24.96	*Cldn22*
Lipid metabolism	A_66_P139646	4.18	62.65	262.86	*Pla2g4c*
	A_55_P2025514	-2.00	65.99	33.15	*Pnpla3*
	A_55_P2182716	-2.29	71.60	31.43	*Adig*
	A_51_P336833	-2.58	11572.62	4501.66	*Fabp4*
	A_51_P259296	-2.96	1718.13	583.30	*Lpl*
	A_55_P2042500	-3.40	365.18	107.90	*Adig*
	A_52_P257812	-3.72	149.53	40.32	*Lpl*
	A_52_P682382	-7.07	2643.35	375.38	*Scd1*
	A_51_P244497	-10.12	251.43	24.92	*Plin1*
	A_55_P2121042	-27.89	38836.43	1397.36	*Pla2g2a*

C

Pathway DOWN [(-/-)/(+/+)]	p-value
PPAR signaling pathway WP2316_69143	2.69E-08
Adipogenesis genes WP447_87026	1.31E-04
Tryptophan metabolism WP79_79761	0.001376
Fatty acid omega oxidation WP33_71721	0.002553
Steroid biosynthesis WP55_89970	0.009068
Retinol metabolism WP1259_89974	0.009565
GPCRs, class A rhodopsin-like WP189_79710	0.014504
Metapathway biotransformation WP1251_69747	0.017514
Statin pathway WP1_73346	0.019018

Supplemental Table 2
Primers for quantitative RT-PCR used in this study.

Primer/probe list for TaqMan Gene Expression Assay

Name	Assay No.
Arg1	Mm00475988_m1
Cd8	Mm01182107_g1
Cd11c/Itgax	Mm00498698_m1
Fcer1a	Mm01295725_m1
Foxp3	Mm00475162_m1
Il1b	Mm00434228_m1
Il6	Mm00446190_m1
Il13	Mm00434204_m1
Il17a	Mm00439618_m1
Krt1	Mm00492992_g1
Krt6a	Mm00833464_g1
Lgr5	Mm00438890_m1
Pla2g2a	Mm00448160_m1
Sox9	Mm00448840_m1
Sox13	Mm00488352_m1
S100a9	Mm00656925_g1
Gapdh	Mouse GAPD (GAPDH) Endogenous Control (Cat# 4352339E)
